# Integrated experimental design and machine learning framework for predicting UV influenced mechanical properties in polyurethane nanodiamond nanocomposites

**DOI:** 10.1038/s41598-026-49606-9

**Published:** 2026-05-18

**Authors:** Markapudi Bhanu Prasad, Abdullah A. Elfar, P. S. Rama Sreekanth, Santosh Kumar Sahu, Borhen Louhichi, It Ee Lee, Qamar Wali

**Affiliations:** 1 School of Mechanical Engineering, VIT-AP University, Besides A.P. Secretariat, Amaravati, 522237 Andhra Pradesh India; 2 Department of Industrial Engineering, College of Engineering, Imam Mohammad Ibn Saud Islamic University (IMSIU), Riyadh, 11432 Saudi Arabia; 3 Engineering Sciences Research Center (ESRC), Deanship of Scientific Research, Imam Mohammad Ibn Saud Islamic University (IMSIU), Riyadh, 11432 Saudi Arabia; 4 Faculty of Artificial Intelligence and Engineering, Multimedia University, Cyberjaya, 63100 Malaysia; 5 Centre for Smart Systems and Automation, COE for Robotics and Sensing Technologies, Multimedia University, Cyberjaya, 63100 Malaysia

**Keywords:** PU, ND, UV, Taguchi, ANOVA, Machine learning, Engineering, Materials science, Nanoscience and technology, Physics

## Abstract

This study investigates the influence of ultraviolet (UV) irradiation on the mechanical performance of nanodiamond (ND) reinforced polyurethane (PU) nanocomposites. The Taguchi method was employed to systematically design the experiments, while analysis of variance (ANOVA) was used to evaluate the statistical significance and percentage contribution of each factor. An L27 orthogonal array was adopted to examine the effects of composition (pure PU, 0.2 wt% PU/ND, and 0.5 wt% PU/ND), UV exposure duration (0, 200, and 400 h), UV irradiation intensity (1.0, 1.20, and 1.40 W/m²), and UV temperature (40, 50, and 60 °C) on tensile strength, Young’s modulus, and hardness. The results indicate that 200 h of UV exposure enhances tensile strength and Young’s modulus for all samples, with the most pronounced improvement observed in the 0.5 wt% PU/ND nanocomposite, whereas hardness decreases with increasing exposure due to UV-induced surface degradation. ANOVA results indicate that composition and UV exposure duration are the most influential parameters, contributing 48.76% and 17.58% to tensile strength and 40.21% and 19.13% to Young’s modulus, respectively. For hardness, UV exposure duration is the dominant factor, contributing 49.6%. Machine learning models, including linear regression, artificial neural networks, and Gaussian process regression, were developed for prediction. Among them, the Gaussian process model showed the highest accuracy with R² values of 0.99, 0.95, and 0.98 for tensile strength, Young’s modulus, and hardness, respectively. These findings highlight the potential of PU/ND nanocomposites for applications in automotive components, robotic parts, and aerospace structures.

## Introduction

Thermoplastic polymers are a class of polymers that can be repeatedly melted and reprocessed without chemical degradation^1^. This characteristic allows them to be melted and reshaped repeatedly, providing versatility in manufacturing processes^2^. Among the various polymer classes, thermoplastic polyurethane (PU) functions as a thermoplastic elastomer, combining the characteristics of plastics along with elasticity. Its segmented molecular structure, consisting of flexible soft segments and rigid hard segments, provides a unique balance of mechanical strength, flexibility, and ease of processability, making it suitable for advanced engineering applications^3–5^. Despite these advantages, pristine polyurethane (PU) exhibits inherent limitations, including relatively low stiffness and moderate mechanical strength^6,7^. To overcome these shortcomings, various nanofillers have been incorporated into pure PU^8,9^. The following are key studies about the influence of the mechanical characteristics of PU nanocomposites. Chen et al.^10^. Identified that Polyurethane reinforced with mini-sized graphene at 3 wt% enhanced the tensile strength of 81% and modulus of 126.7%. The addition of 0.05 wt% graphene to the PU matrix resulted in an increase in tensile strength by 26% and in Young’s modulus by 21%, respectively^11^. Adding Halloysite Nanotubes (HNTs) from (0–10 wt%) enhanced tensile strength by 30.4% and tensile modulus by 47.2% at 8 wt% compared to pure PU^12^. The addition of 5 wt% nanosilicon increased compressive strength, tensile strength, and shear strength by 29.4%, 257.6%, and 202.1%, compared to virgin pure PU^13^. PU reinforced with CNT exhibited significant enhancement in tensile strength (15.4 MPa) and elongation at break (420%)^14^. The tensile strength of GO/TPU composites improved with GO concentration, obtaining a maximum improvement of 194% at 80 wt% GO compared to pure TPU^15^. Adding 1% MWCNTs to PU increased ultimate tensile strength and modulus by 21% and 25%, respectively^16^. HNTs addition in the TPU with 10 wt% witnessed an increase in tensile modulus and hardness to 4.91 GPa and 76.57 HV, respectively, compared with pure TPU^17^. The inclusion of polyurethane with 0.02 wt% graphene oxide (GO) improved its tensile strength by 60%, respectively^18^—the incorporation of 5.5 wt% NDs into PU micro/nanofiber membranes enhanced Young’s modulus, tensile strength, and elongation at break by 29%, 105%, and 66%^19^. Polyurethane reinforced with MWCNTs at 10 wt% enhanced modulus of elasticity and hardness by 124% and 53%, respectively^20^. adding 0.1–0.5 wt% ND, tensile strength, Young’s modulus, and hardness were enhanced by 114%, 11%, and 21%, respectively^21^—the addition of 0.5 wt% MXene-to-PU increased tensile modulus, strength and hardness, enhanced by 22% and 281% and 19%^22^.

Previous studies have extensively explored polyurethane (PU) reinforced with nanofillers such as CNTs, graphene, GO, nanosilica, MXene, and HNTs, demonstrating significant improvements in mechanical properties. However, these fillers often involve trade-offs, particularly under UV exposure, where issues such as agglomeration, interfacial degradation, and limited UV shielding can adversely affect long-term performance^23,24^. In contrast, nanodiamond (ND) offers a unique combination of high hardness, chemical inertness, and superior resistance to photo-degradation, making it a promising alternative for enhancing both mechanical performance and UV stability^25^. Despite these advantages, the application of ND in PU, particularly under UV ageing conditions, remains largely unexplored. Furthermore, existing studies primarily focus on experimental observations, with limited integration of data-driven modelling approaches to understand and predict composite behaviour. In this manner, the current study addresses this gap by combining Taguchi design of experiments with machine learning techniques to systematically analyse and optimise the performance of ND-reinforced PU under UV exposure. This integrated approach enables a deeper understanding of the mechanical trade-offs and durability, establishing the novelty of the present work.

## Materials and methods

### Materials

**Fig. 1:**
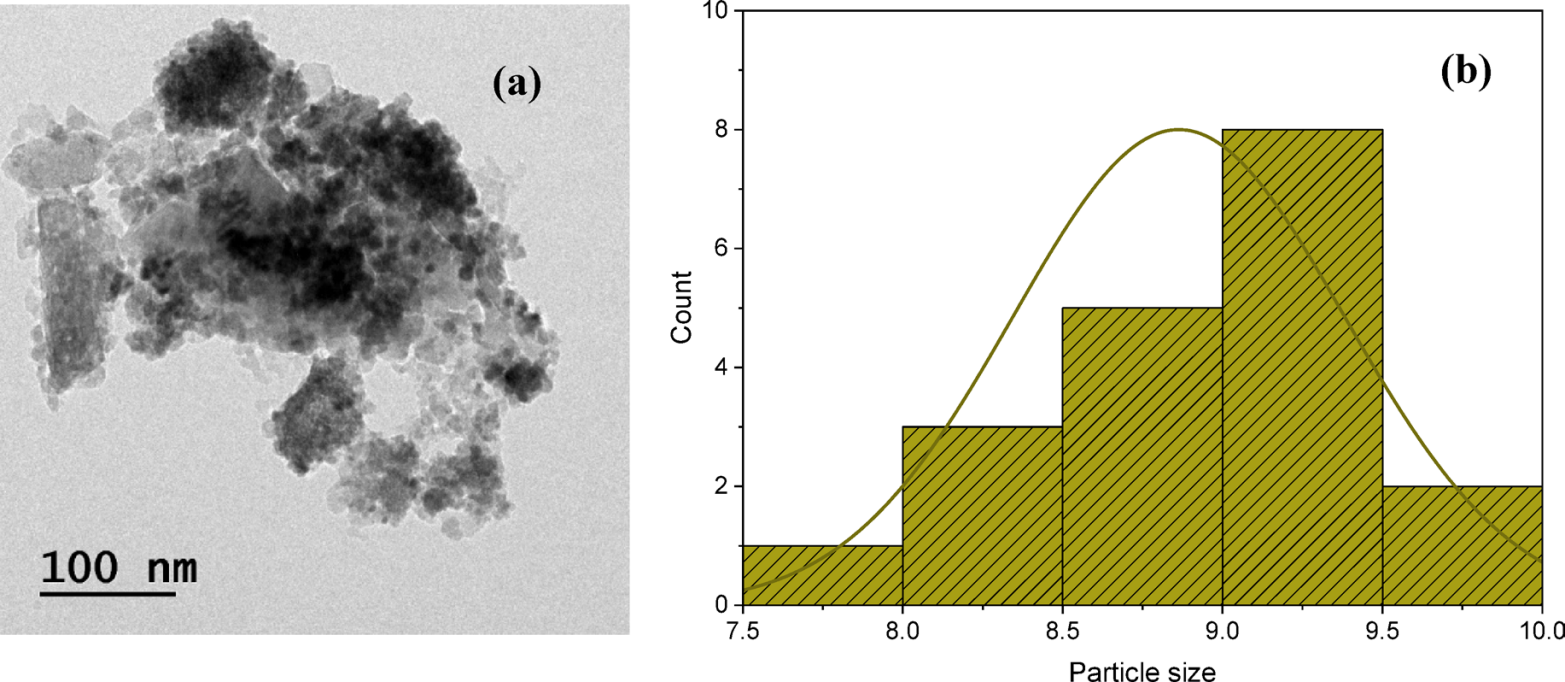
(**a**) TEM image of ND; (**b**) particle size of ND.

Polyurethane (PU) granules were acquired from SMP Technologies Inc., Tokyo, Japan. Nanodiamond (ND) nanofillers are acquired from Nano Research Element Inc., Delhi, India. The PU pellets have diameters varying from 7 to 8 mm, with a density of 834 kg/m^3^, and a glass transition temperature (T_g_) of 65 °C. The ND fillers were distributed as a fine powder with particle sizes < 10 nm. These nanofillers possessed a density of 3180 kg/m³, purity above 99%, and a specific surface area (A_s_) of approximately 350 m²/g. The morphology of the provided ND particles was investigated using Transmission Electron Microscopy (FEI Tecnai, G2 T20, 200 KeV). The TEM analysis (Fig. 1a) revealed that the nanodiamonds exhibit a characteristic dot-like structure. The particle size analysis, as illustrated in Fig. 1b, revealed that the average particle size was less than 10 nm.

### Fabrication of samples

**Fig. 2:**
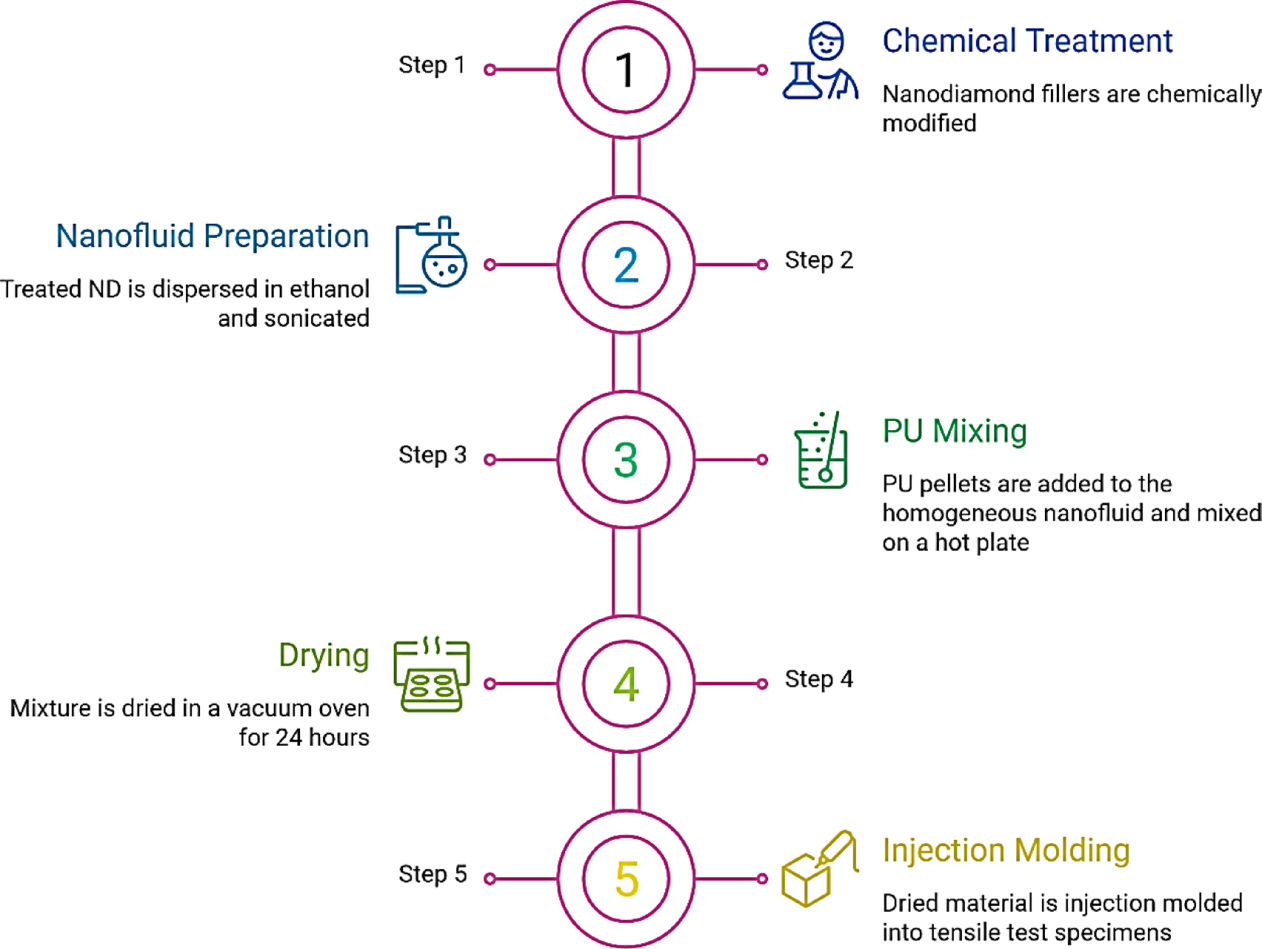
Fabrication steps.

Polyurethane nanodiamond nanocomposite (PU/ND) specimens containing 0.2 wt% & 0.5 wt% ND were fabricated. The fabrication steps are illustrated in Fig. 2. Initially, ND particles were chemically functionalized with a mixed-acid treatment (98% sulfuric acid + 70% nitric acid at a 3:1 volume ratio). The treated ND was washed, filtered, and dried at 80 °C to obtain carboxylated ND. The required amount of modified ND was then dispersed in ethanol through sonication to achieve uniform dispersion. Subsequently, PU pellets are added to the suspension and mixed thoroughly on a hot plate to obtain a homogeneous mixture. The mixture is dried in an Oven chamber for 24 h to remove the solvent and moisture. Finally, the evaporated material was processed by injection moulding to produce tensile test specimens in accordance with ASTM D638. The fabricated samples were designated as virgin PU, 0.2 wt% ND/PU, & 0.5 wt% ND/PU.

### UV Irradiation

UV radiation tests were carried out using an Apple Electronics Tabletop Accelerated Weathering Tester (AWT). The samples were exposed under different test conditions, including UV Irradiation (1.0, 1.20, 1.40) W/m2 with durations of (0, 200, 400) hours, UV Temperature (40, 50, 60) °C, and a Wavelength of 340 nm under ambient air.

### Tensile test

Tensile properties were evaluated using an H10KL Universal Testing Machine by Tinius Olsen India Pvt. Ltd., Uttar Pradesh, India. The experiments were carried out following the procedure specified in ASTM D638. Testing was conducted under ambient laboratory conditions with the crosshead moving at 2 mm/min. For reliability, three specimens from each composition were tested, and the measurements were repeated for three trials.

### Hardness test

The hardness was measured with a Vickers hardness tester. An indentation was made on the surface of the specimen by a diamond indenter at a load of 1 kg for a dwell time of 20 s. For each sample, measurements were taken at three different locations to ensure accuracy and repeatability. A Vickers Hardness Number (HV) was derived using Eq. (1)^27^.

1 $$\:HV=1.889\:\frac{P}{{d}^{2}}$$

Where P is the applied force (Newton), and d is the average length of the diagonal of the indentation in micrometres.

### Design of experiments (DOE) by Taguchi

The Taguchi method is a statistical approach to design, primarily used in engineering and industrial research. The method reduces the number of experimental runs required to achieve valid statistical outcomes^28^. This study selected four factors that assess the mechanical properties of the composites, and Minitab version 21 software was used to evaluate the results. The process parameters are (i) Composition, (ii) UV Duration Time, (iii) UV Irradiation, and (iv) UV Temperature. Table 1 shows the three assessment levels (L1, L2, and L3) used to evaluate each parameter. An experimental design with four process parameters at three levels required (3^4^ = 81) experimental runs to study all possible factor combinations. However, conducting 81 experiments is time-consuming and resource-intensive. Therefore, the Taguchi L27 design of orthogonal array was chosen for the experimental design. This array reduces the number of required experiments to 27 runs, as illustrated in Table 2^29,30^.

**Table 1:** Level of process parameters.

Sl. No.	Factors	L-1	L-2	L-3
1	Composition (wt%)	0	0.2	0.5
2	UV Duration Time(Hours)	0	200	400
3	UV Irradiation(W/m^2^)	1.0	1.20	1.40
4	UV Temperature(°C)	40	50	60

**Table 2:** Taguchi L27 orthogonal array.

Exp. No.	Composition(wt%)	UV Duration Time(Hours)	UV Irradiation (W/m^2^)	UV Temperature(°C)
1	0	0	1	40
2	0	0	1	40
3	0	0	1	40
4	0	200	1.2	50
5	0	200	1.2	50
6	0	200	1.2	50
7	0	400	1.4	60
8	0	400	1.4	60
9	0	400	1.4	60
10	0.2	0	1.2	60
11	0.2	0	1.2	60
12	0.2	0	1.2	60
13	0.2	200	1.4	40
14	0.2	200	1.4	40
15	0.2	200	1.4	40
16	0.2	400	1	50
17	0.2	400	1	50
18	0.2	400	1	50
19	0.5	0	1.4	50
20	0.5	0	1.4	50
21	0.5	0	1.4	50
22	0.5	200	1	60
23	0.5	200	1	60
24	0.5	200	1	60
25	0.5	400	1.2	40
26	0.5	400	1.2	40
27	0.5	400	1.2	40

### Signal-to-noise ratio (S/N ratio)

A Taguchi design technique transforms the experimental data into a Signal-to-Noise (S/N) ratio, which serves as the objective metric for optimisation. The S/N ratio quantifies the trade-off between desirable performance (the signal) and undesirable variance (the noise). This S/N ratio enables better verification of results and helps identify the right process parameters. The Taguchi methodology emphasises reducing deviations from the desired response by using an S/N ratio to evaluate performance consistency and quality stability. The S/N ratio evaluation includes three main categories: “smaller-the-better,” “larger-the-better,” and “nominal-the-best” evaluation methods. The “larger-the-better” criterion was used in this study to increase the mechanical performance of the materials. The equivalent S/N ratio for this category is represented in Eq. (2)^31^.

2 $$\:\frac{S}{N}=-10{\mathrm{log}}_{10}\left(\frac{1}{n}{\sum\:}_{i=1}^{n}\frac{1}{{x}_{i}^{2}}\right)$$

Where $\:{x}_{i}$=true data points, and n = number of measurements.

### Analysis of variance (ANOVA)

Analysis of Variance (ANOVA) is a statistical method for examining how multiple input variables affect experimental outcomes. ANOVA tests show how each factor contributes to the overall results by partitioning the total variation into separate components. The ANOVA was performed using Minitab version 21 at the 95% confidence level^32^. The present study employed ANOVA to examine the effects of four parameters on the mechanical properties of PU/ND composites: composition, UV duration, UV irradiation, and UV Temperature. Using an L27 orthogonal array permits a reduction in the number of experimental runs while maintaining equally effective and accurate statistical analyses under the same standards.

### Machine learning

Machine learning (ML) was utilised to evaluate tensile strength, Young’s modulus, and hardness of PU/ND nanocomposites. Three algorithms were used: Linear Regression, Artificial Neural Network (ANN), and Gaussian Process Regression (GPR). The models were trained using data obtained from controlled UV irradiation experiments. The processing parameters were used as input variables, while the measured mechanical properties were taken as outputs. After training and testing models, they were applied to the experimental dataset to determine suitable processing conditions. The experimental data were processed using Python (version 3.9) on Google Colab. Model efficiency was assessed using 5-fold cross-validation. In this approach, the dataset was divided into five equal parts^31,32^. Models were trained utilising four parts, and their predictions were evaluated on the final portion. This step was repeated 5 times, with each subset serving as the validation set once. The average across all five runs provided a balanced measure of model performance. Model accuracy was measured using standard evaluation metrics: mean squared error (MSE), root mean squared error (RMSE), coefficient of determination (R²), mean absolute error (MAE), and mean absolute percentage error (MAPE)^33^. The mathematical expressions for these metrics are given in Eqs. (3)–(7)^33,34^.

3 $$\:{R}^{2}=1-\:\:\:\frac{\sum\:_{i=1}^{n}{({c}_{i}-{\stackrel{-}{c}}_{i}\:)}^{2}}{\sum\:_{i=1}^{n}{({c}_{i}-\stackrel{-}{c}\:)}^{2}}$$

4 $${\mathrm{MSE}}=\:\:\:\frac{1}{n}\sum\:_{i=1}^{n}{({c}_{i}-{\stackrel{-}{c}}_{i})}^{2}$$

 

5 $${\mathrm{RMSE}}=\:\:\sqrt{MSE}$$

 

6 $${\mathrm{MAE}}\:\:\frac{1}{n}\sum\:_{i=1}^{n}\left|{c}_{i}-{\stackrel{-}{c}}_{i}\right|$$

7 $${\mathrm{MAPE}}=\:\frac{1}{n}{\sum\:}_{i=1}^{n}\left|\frac{{c}_{i}-{\stackrel{-}{c}}_{i}}{{c}_{i}}\right|\times100$$

Where *n* is the number of data points in the dataset, *c*_*i*_ is the experimental values of the response variable for the ith observation, ${\stackrel{-}{c}}$ is the mean of all actual observed values, and ${\stackrel{-}{c}}_{i}$ is the predicted values of the response variable for the *i*^th^ observation.

#### Linear regression

Linear regression is a type of regression analysis that determines how a dependent variable depends on multiple independent variables by fitting a straight line to the data. Multiple linear regression requires more than one predictor variable, whereas linear regression uses only one. The model parameters are determined by minimising the total squared error between predicted outcomes and actual results, assuming that input variables have a linear relationship with output variables. The method predicts numerical results and serves as a fundamental component of statistical learning theory, as described in Eqs. (8–9)^35^.

8 $$\:\stackrel{-}{Z\:}=\:{Q}_{0}+\:{Q}_{1}{Y}_{1}+{Q}_{2}{Y}_{2}+{Q}_{3}{Y}_{3}+{Q}_{4}{Y}_{4}+{Q}_{5}{Y}_{5}$$

$\:\mathrm{w}\mathrm{h}\mathrm{e}\mathrm{r}\mathrm{e}\:\mathrm{Z}=\mathrm{p}\mathrm{r}\mathrm{e}\mathrm{d}\mathrm{i}\mathrm{c}\mathrm{t}\mathrm{e}\mathrm{d}\:\mathrm{r}\mathrm{e}\mathrm{s}\mathrm{p}\mathrm{o}\mathrm{n}\mathrm{s}\mathrm{e},$ *Y*_*1*,_ *Y*_*2*_, *Y*_*3*,_ *Y*_*4*_, *Y*_*5*_ are the Composition, UV Duration Time, UV Irradiation, UV Temperature; *Q*_*1*_, *Q*_*2*_, *Q*_*3*_, *Q*_*4*_, *Q*_*5*_ are model coefficients; _0_ is the intercept.

Estimation of Regression Coefficients:

9 $$\:\stackrel{-}{Q\:}={\left({P}^{T\:}P\right)}^{-1}\:\:{P}^{T\:}Y$$

P is an input variable matrix, while Y is a response output that includes the Tensile strength, Young’s modulus, and hardness.

#### Artificial neural network

An artificial neural network (ANN) represents a digital computer simulation containing many processing nodes, or artificial neurons, that operate through their interconnections. It normally consists of three main components: an input layer, one or more hidden layers, and an output layer. The neurons in each layer establish connections via weighted links, whose weights determine the strength of signals transmitted between neurons. The neurons in the hidden layer process incoming signals from the input layer, multiply them by their respective weights, and then add a bias term. ANN models can effectively capture complex nonlinear interactions between both dependent and independent variables. The ANN model was implemented as a multilayer perceptron (MLP) regressor with a base architecture comprising a single hidden layer of 50 neurons. The network was trained for 800 epochs using the ReLU activation function and the Adam optimiser, with a random seed of 42 to ensure reproducibility. This capability makes ANN suitable for predicting material characteristics, including tensile strength, Young’s modulus, and hardness. The hidden neuron’s output (*H*_*j*_​) is expressed in Eq. (10)^36^.

10 $$\:{H}_{j}=f\left({\sum\:}_{i=1}^{n}{U}_{ij}{Z}_{i}+{a}_{j}\right)$$

Where *H*_j_ is the output of the *j*^*th*^ hidden neuron, *f* represents the stimulation factor, *n* represents the total number of input parameter variables, *U*_*ij*_ represents the weights connecting the *i*^th^ input to the *j*^*th*^ hidden neuron, *a*_*j*_ is the bias term, *Z*_*i*_ are input variables such as composition, UV duration time, UV Irradiation, and UV temperature.

#### Gaussian process regression analysis

Gaussian Process Regression (GPR) is a machine learning method that models complex nonlinear relationships between input and output variables through its non-parametric, probabilistic modelling capabilities. GPR predicts output values by combining the prior distribution over functions with observed data, assuming a joint Gaussian distribution. GPR provides both predicted values and uncertainty estimates, which makes it suitable for predicting material characteristics such as tensile strength, Young’s modulus, and hardness. This model employs a composite kernel (Constant Kernel × Radial Basis Function (RBF) kernel) with hyperparameters optimised by maximising the log marginal likelihood during training. Additionally, all input and output data were standardised prior to model training to improve numerical stability and performance. The GPR is characterised by its mean and covariance functions, as shown in Eq. (11)^37^.

11 $$\:f\left(z\right){\sim}GP\left(m\left(z\right),k\left(z,{z}^{{\prime\:}}\right)\right)$$

Where f(z) is the function value at input z, *m(z)* is the mean function and *k (z*, $\:{z}^{{\prime\:}}$*)* is the covariance (kernel) function that quantifies the similarity between input points z and z′.

#### Interpretation of machine learning by using SHAP analysis

The Shapley Additive Explanations (SHAP) evaluation improves the interpretability of machine learning models by showing how input features influence predictions of mechanical characteristics. SHAP is a game-theory-based interpretation approach that assigns relevance scores to each input feature based on its contribution to the model’s prediction. In SHAP analysis, the most important processing parameters are unanimously identified from all datasets. which generates complete understanding of the primary factors that determine all model outputs^38,39^.

## Results and discussion

### Tensile test

**Fig. 3:**
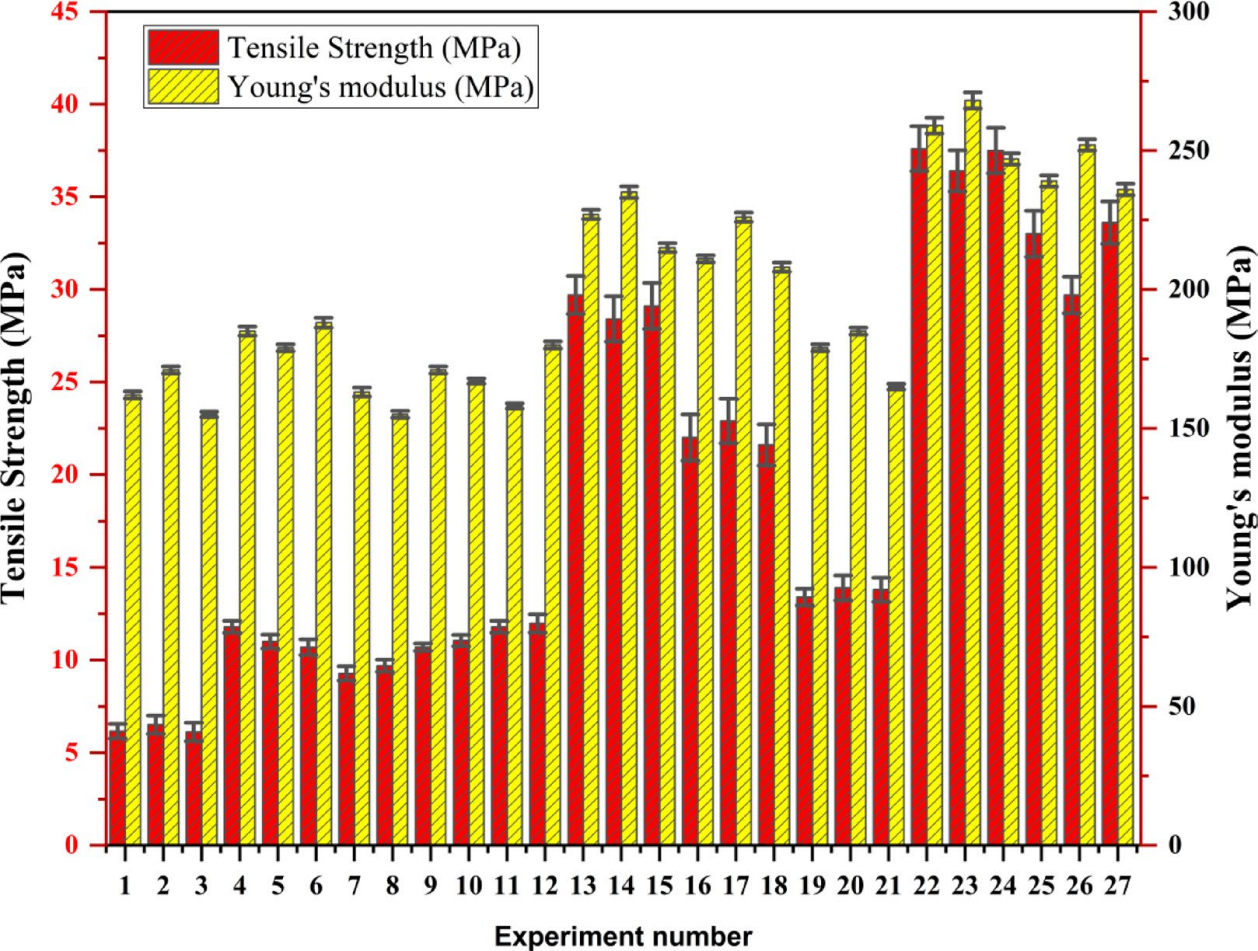
Tensile strength and Young’s modulus vs. experiment number.

Figure 3 presents the tensile strength and Young’s modulus results from 27 experimental tests conducted using an L27 orthogonal array. The tensile strength ranges from 6.12 MPa to 37.6 MPa, whereas Young’s modulus ranges from 155 MPa to 268 MPa, showing a strong dependence on composition, UV duration, UV irradiation, and UV temperature. The specimens tested at 0 h in Experiments 1–3, 10–12, and 19–21 exhibit the lowest tensile strength and modulus values for the unaged pure and its composite. The results from Experiments 4–6,13–15, and 22–24, at 200-hour UV exposure, show an increase in both tensile strength & Young’s modulus, particularly at 0.5 wt% PU/ND composite. This improvement is ascribed to UV-induced photo-crosslinking in the polyurethane matrix and effective reinforcement^40^. Besides, the high surface area of nanodiamond provides strong interfacial bonding and serves as a crosslinking surface, thereby minimising the polymer chain mobility while increasing load transmission^41^. As a result, the material exhibits higher stiffness and strength before long-term exposure to photo-oxidative chain scission. Experiments 7–9, 16–18, and 25–27 at a longer UV exposure of 400 h show a decrease in tensile and modulus characteristics. This deterioration is due to photo-oxidative breakdown of the polyurethane matrix. Longer UV Exposure causes chain breakage, which degrades cross-linked structures, induces surface cracking, and deteriorates the filler-matrix bond^42^. These findings demonstrate that the addition of nanodiamond substantially improves the tensile strength and Young’s modulus.

#### Hardness Testing

**Fig. 4:**
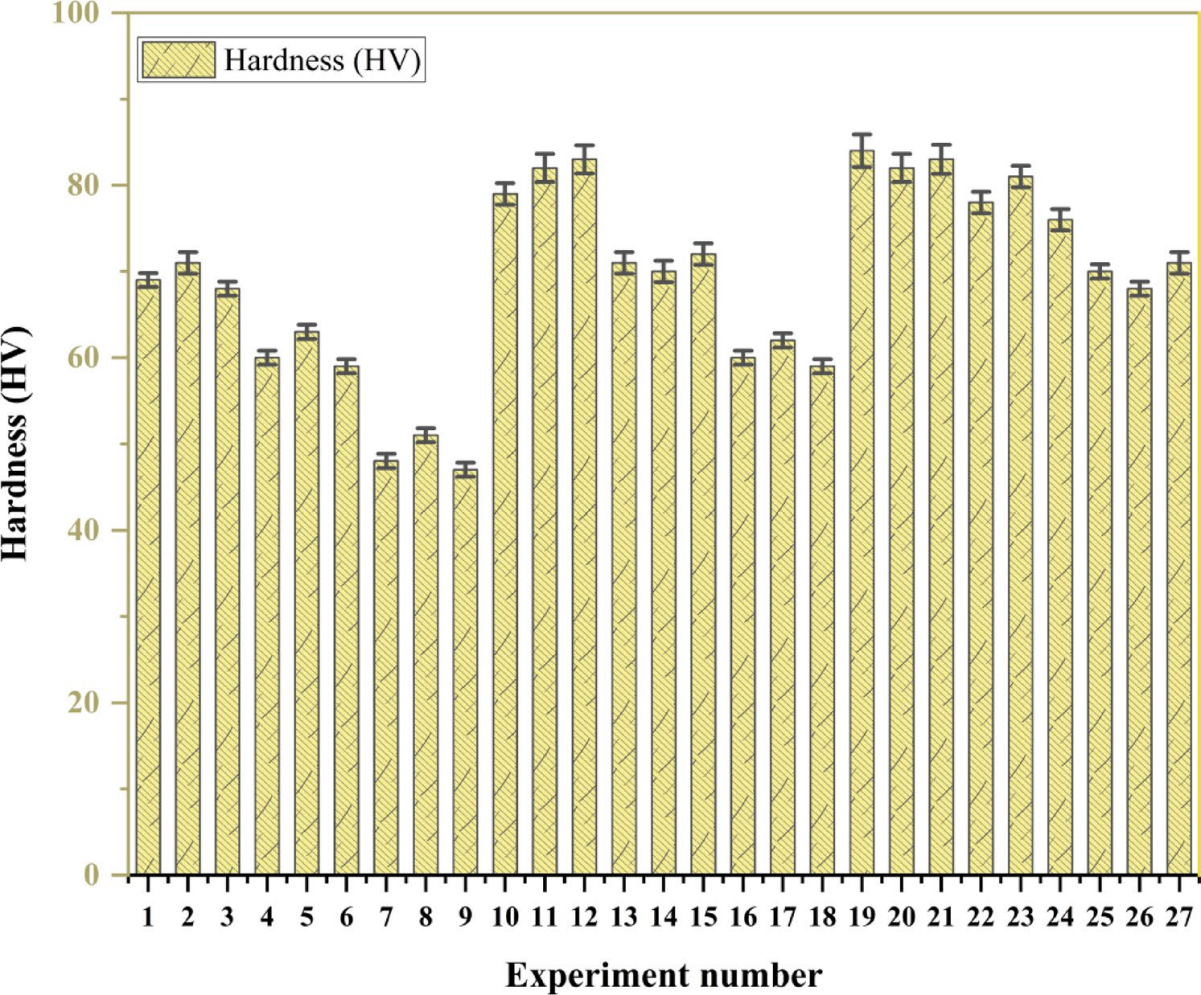
Hardness vs. experiment number.

Microhardness testing was performed to study how different ultraviolet conditions affected the mechanical properties of polyurethane composites containing nanodiamond (ND) reinforcement. The Taguchi L27 orthogonal array was employed to find the best combination of UV factors, and the results are presented graphically in Fig. 4. At 0 h of UV exposure, Experiment (1, 10 and 19) samples with 0.5 wt% PU/ND had higher hardness values (69–84 HV) than pure PU due to the inherent stiffness of ND particles, larger surface area and high interfacial interaction between ND and PU matrix. At 200 h UV exposure (Experiments 4–6, 13–15, and 22–24), all compositions show a considerable loss in hardness. A similar trend was observed at 400 h of UV exposure (Experiments 7–9, 16–18, 25–27), resulting in a significant loss of hardness due to photo-oxidation degradation of the matrix via chain scission, surface embrittlement, and weakening of the filler-matrix interfaces^43^. However, ND-reinforced samples retain higher values than pure PU.

### Statistical evaluation

#### Taguchi examination

The Taguchi method used experimental data for tensile strength, Young’s modulus, and hardness to determine S/N ratios, as presented in Table 3. The following sections explain how to use the Taguchi method to determine optimal process parameters for various mechanical properties.

**Table 3:** Taguchi L27 design tests data for tensile strength, Young’s modulus, and Hardness, along with their S/N ratios.

Ex. no	Composition (wt%)	UV Duration Time (Hours)	UV Irradiation (W/m^2^ )	UV Temperature (^0^C)	Tensile Strength (MPa)	Young’s modulus (MPa)	Hardness (HV)	S/*N* ratios (Tensile Strength)	S/*N* ratios (Young’s modulus)	S/*N* ratios (Hardness)
1	0.0	0	1.0	40	6.16	162	69	15.9262	44.2050	36.8147
2	0.0	0	1.0	40	6.51	171	71	15.9262	44.2050	36.8147
3	0.0	0	1.0	40	6.12	155	68	15.9362	44.2050	36.8147
4	0.0	200	1.2	50	11.80	185	60	20.9365	45.2909	35.6490
5	0.0	200	1.2	50	11.00	179	63	20.9365	45.2909	35.6490
6	0.0	200	1.2	50	10.70	188	59	20.9365	45.2909	35.6490
7	0.0	400	1.4	60	9.28	163	48	19.8612	44.2228	33.7291
8	0.0	400	1.4	60	9.70	155	51	19.8612	44.2228	33.7291
9	0.0	400	1.4	60	10.70	171	47	19.8612	44.2228	33.7291
10	0.2	0	1.2	60	11.06	167	79	21.2834	44.4864	38.1996
11	0.2	0	1.2	60	11.80	158	82	21.2834	44.4864	38.1996
12	0.2	0	1.2	60	11.98	180	83	21.2834	44.4864	38.1996
13	0.2	200	1.4	40	29.70	227	71	29.2635	47.0518	37.0234
14	0.2	200	1.4	40	28.40	235	70	29.2635	47.0518	37.0234
15	0.2	200	1.4	40	29.10	215	72	29.2635	47.0518	37.0234
16	0.2	400	1.0	50	22.00	211	60	26.9063	46.6318	35.6056
17	0.2	400	1.0	50	22.90	226	62	26.9063	46.6318	35.6056
18	0.2	400	1.0	50	21.60	208	59	26.9063	46.6318	35.6056
19	0.5	0	1.4	50	13.40	179	84	22.7311	44.8964	38.3803
20	0.5	0	1.4	50	13.90	185	82	22.7311	44.8964	38.3803
21	0.5	0	1.4	50	13.80	165	83	22.7311	44.8964	38.3803
22	0.5	200	1.0	60	37.60	259	78	31.4002	48.2178	37.8700
23	0.5	200	1.0	60	36.40	268	81	31.4002	48.2178	37.8700
24	0.5	200	1.0	60	37.50	247	76	31.4002	48.2178	37.8700
25	0.5	400	1.2	40	33.00	239	70	30.0911	47.6778	36.8563
26	0.5	400	1.2	40	29.70	252	68	30.0911	47.6778	36.8563
27	0.5	400	1.2	40	33.60	236	71	30.0911	47.6778	36.5863

##### Tensile strength

The means and signal-to-noise ratios are shown in Tables 4 and 5. Demonstrate how tensile strength varies with each factor’s value, and show how changes in parameters affect the test result. The delta values, which measure each factor’s relative strength. The composite material had the most significant influence, with a delta value of 18.548 for the mean and 9.17 for the signal-to-noise ratio. In contrast, UV Duration time had a delta of 15.274 for the mean and 7.22 for the S/N ratio. The UV temperature and UV irradiation processes have only a low impact on tensile strength.

**Table 4:** means response.

Level	Composition	UV duration Time	UV Irradiation	UV Temperature
1	9.108	10.526	21.866	22.477
2	20.949	25.800	18.293	15.678
3	27.656	21.387	17.553	19.558
Delta	18.548	15.274	4.312	6.799
Rank	1	2	4	3

**Table 5:** S/N Ratios response.

Level	Composition	UV Duration Time	UV Irradiation	UV Temperature
1	18.91	19.98	24.74	25.09
2	25.82	27.20	24.10	23.52
3	28.07	25.62	23.95	24.18
Delta	9.17	7.22	0.79	1.57
Rank	1	2	4	3

**Fig. 5:**
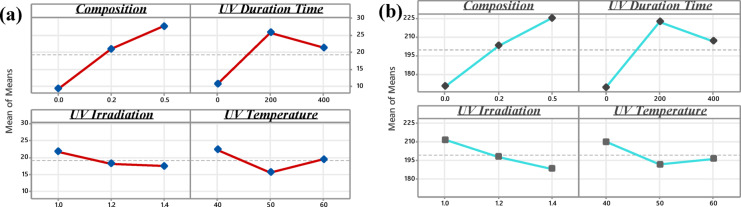
Mean effect plots for (**a**) tensile strength (**b**) Young’s modulus.

In Fig. 5a shows the, Mean impact plot for tensile strength confirms that the composition and UV exposure time are the most important factors that affect tensile strength. In contrast, UV temperature and UV irradiation have only a minimal impact. The mean response rises with a composite change but decreased with increasing UV Duration Time. The tensile strength increases notably with the addition of 0.5 wt% PU/ND composite material demonstrates that the nanodiamonds act as reinforcing agents. Figure 5b, Mean effects plot for Young’s modulus confirms that composition is the most significant factor across all factors. The remaining variables show a minor effect. The composition and UV duration are identified as the major factors determining both tensile strength and Young’s modulus, while UV temperature and UV irradiation exhibit minimal impact on the mechanical properties.

##### Young’s modulus

**Fig. 6:**
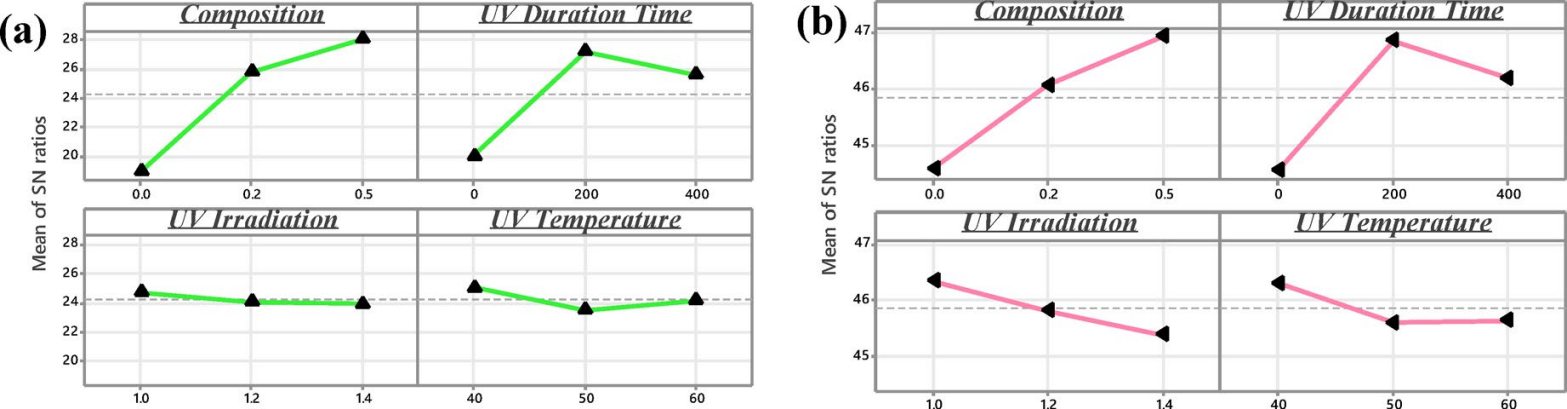
Mean of S/N ratios for (**a**) tensile strength, (**b**) Young’s modulus.

Tables 6 and 7 show how the response varies with different factor levels, including both the mean response and signal-to-noise ratio outcomes. The Delta values show that the composition is the most influential factor in both mean (55.7) and signal-to-noise ratio (2.36). The UV Duration is the second most impactful factor, with a delta of 53.4 for the mean and 2.32 for the signal-to-noise ratio. In contrast, UV Irradiation and UV Temperature are less influential on the Young’s modulus. The Taguchi experimental design was applied to identify the optimal control parameters, through S/N ratios that evaluate how parameters withstand noise interference as demonstrated in Fig. 6a. The S/N ratio examination identifies the composition and UV Duration time as the most dominant factors, whereas UV Irradiation and UV Temperature are the minimal impact. Figure 6b shows that both composition and UV Duration time have a strong impact on the S/N ratios. The effects of UV Irradiation and UV Temperature have a fractional influence on Young’s modulus.

**Table 6:** means response.

Level	Composition	UV duration Time	UV Irradiation	UV Temperature
1	169.9	169.1	211.9	210.2
2	203.0	222.6	198.2	191.8
3	225.6	206.8	188.3	196.4
Delta	55.7	53.4	23.6	18.4
Rank	1	2	3	4

**Table 7:** S/N ratios response.

Level	Composition	UV duration Time	UV Irradiation	UV Temperature
1	44.57	44.53	46.35	46.31
2	46.06	46.85	45.82	45.61
3	46.93	46.18	45.39	45.64
Delta	2.36	2.32	0.96	0.71
Rank	1	2	3	4

##### Hardness

**Fig. 7:**
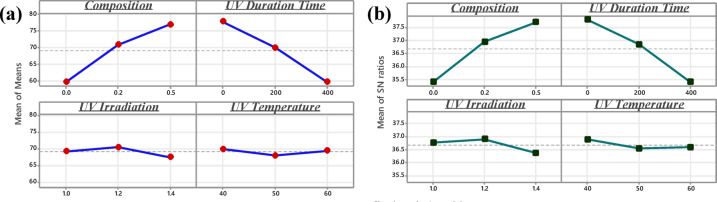
(**a**) Mean of Means, (**b**) Mean of S/N ratios for hardness.

Tables 8 and 9 show the influence of hardness through the mean values and the signal-to-noise (S/N) ratios. These results indicate how variations in processing conditions across different levels of each parameter affect the hardness. The delta value represents the relative impact of each factor. Among the tested parameters, the UV duration time demonstrates the strongest impact, with delta mean and S/N ratio values of 18.33 & 2.40. Composition follows with delta mean and S/N ratio values of 17.44 and 2.30 respectively. The influence of UV irradiation and UV temperature on hardness is much smaller than that of these two factors. Figure 7a presents the Mean effects plot for hardness. The plot indicates that UV duration and composition strongly affect hardness, whereas the effects of UV irradiation and UV temperature are limited. A noticeable increase in hardness is observed when the 0.5 PU/ND composite is used without UV exposure (0 h). Figure 7b shows the Mean effects plot for the hardness S/N ratio. The same trend appears in this plot, where UV duration time and composition have the strongest influence, and UV irradiation and UV temperature show weaker effects.

**Table 8:** Response table of means.

Level	Composition	UV Duration Time	UV Irradiation	UV Temperature
1	59.56	77.89	69.33	70.00
2	70.89	70.00	70.56	68.00
3	77.00	59.56	67.56	69.44
Delta	17.44	18.33	3.00	2.00
Rank	2	1	3	4

**Table 9:** Response table for S/N Ratio.

Level	Composition	UV Duration Time	UV Irradiation	UV Temperature
1	35.40	37.80	36.76	36.90
2	36.94	36.85	36.90	36.54
3	37.70	35.40	36.38	36.60
Delta	2.30	2.40	0.52	0.35
Rank	2	1	3	4

#### Analysis of variance (ANOVA)

##### Tensile strength

Tensile test data obtained from the Taguchi L27 orthogonal array were analysed using Analysis of Variance (ANOVA) to examine the influence of composition, UV duration time, UV irradiation, and UV temperature. The results in Table 10 show that composition accounts for the largest share of the variation in tensile strength (48.76%), followed by UV duration (17.58%). Interaction effects were also observed, with the combination of composition and UV irradiation accounting for 23.16%, and the interaction between UV duration and UV irradiation contributing 3.84%. The other factors all contribute below 10%. These findings demonstrate that the composite composition plays the major role in improving the tensile strength of PU–ND nanocomposites. A regression model was used to estimate tensile strength, and the model indicates strong agreement with the experimental results. The coefficient of determination (R²) is 98.91%, while the adjusted R² value is 98.51%, showing that the model explains most of the variation in tensile strength A Linear Regression model was constructed to establish the relationship between the processing variables. —composition (C), UV duration time (UDT), UV irradiation (UI), and UV temperature (UT)—and tensile strength. The equation of predicting for all samples is given in Eq. (12).

12 $$\begin{gathered}{\text{Tensile Strength (MPa)}}=-48.47+453.9(C)+0.1502{\mathrm{(UDT)}}+95.35{\mathrm{(UI)}}\hfill \\-1.0217{\mathrm{(UT)}}-0.0227-352.7{\mathrm{(C)}}^*{\mathrm{(UI)}}-0.1324{\mathrm{(UDT)}}^*{\mathrm{(UI)}}\end{gathered}$$

**Table 10:** Tensile strength variance analysis.

Source	DF	Seq SS	Contribution	Adj SS	Adj MS	F-Value	P-Value
Composition	1	1471.85	48.76%	880.440	880.440	510.40	0.000
UV Duration Time	1	530.84	17.58%	131.611	131.611	76.30	0.000
UV Irradiation	1	83.68	2.77%	355.804	355.804	206.26	0.000
UV Temperature	1	38.34	1.27%	405.875	405.875	235.29	0.000
Composition*UV Duration Time	1	46.18	1.53%	3.348	3.348	1.94	0.180
Composition*UV Irradiation	1	699.25	23.16%	811.143	811.143	470.23	0.000
UV Duration Time*UV Irradiation	1	115.94	3.84%	115.940	115.940	67.21	0.000
Error	19	32.77	1.09%	32.775	1.725		
Total	26	3018.85	100.00%				

R-sq = 98.91 %  R-sq(adj) = 98.51 %

**Fig. 8:**
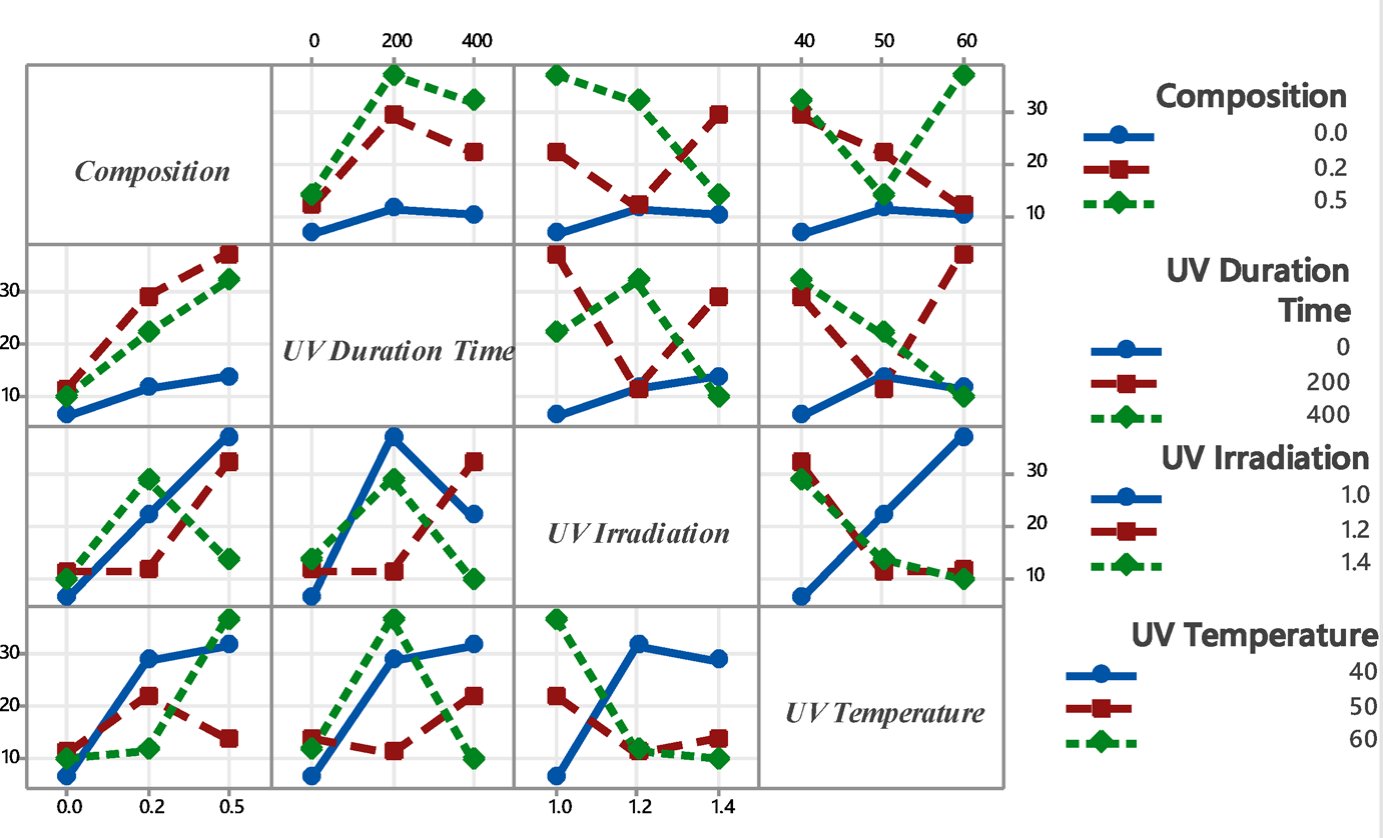
Tensile strength interaction graphs.

The interaction plot shows the tensile strength of the polyurethane/nanodiamond (PU/ND) nanocomposite demonstrates how processing factors interact with each other to determine the material’s mechanical characteristics. The plot indicates that tensile strength is substantially reliant on nanodiamond content, with a significant improvement as composition increases from 0.2 to 0.5 wt% across all UV exposure conditions. This improvement is certainly owing to the strong reinforcing effect of nanodiamonds, which improves stress transmission, stiffness and interfacial bonding with in the polymer matrix.in contract prolonged UV exposure leads to a decrease in tensile strength.as the UV duration rises from 200 to 400 h the tensile strength decreases due to degradation such as chain scission and oxidation and micro crack development which leads to increased brittleness. The interaction plot demonstrates that nanodiamonds play a vital role in enhancing tensile strength requires control of several processing factors are used to ensure optimal mechanical performance as depicted in the Fig. 8.

**Fig. 9:**
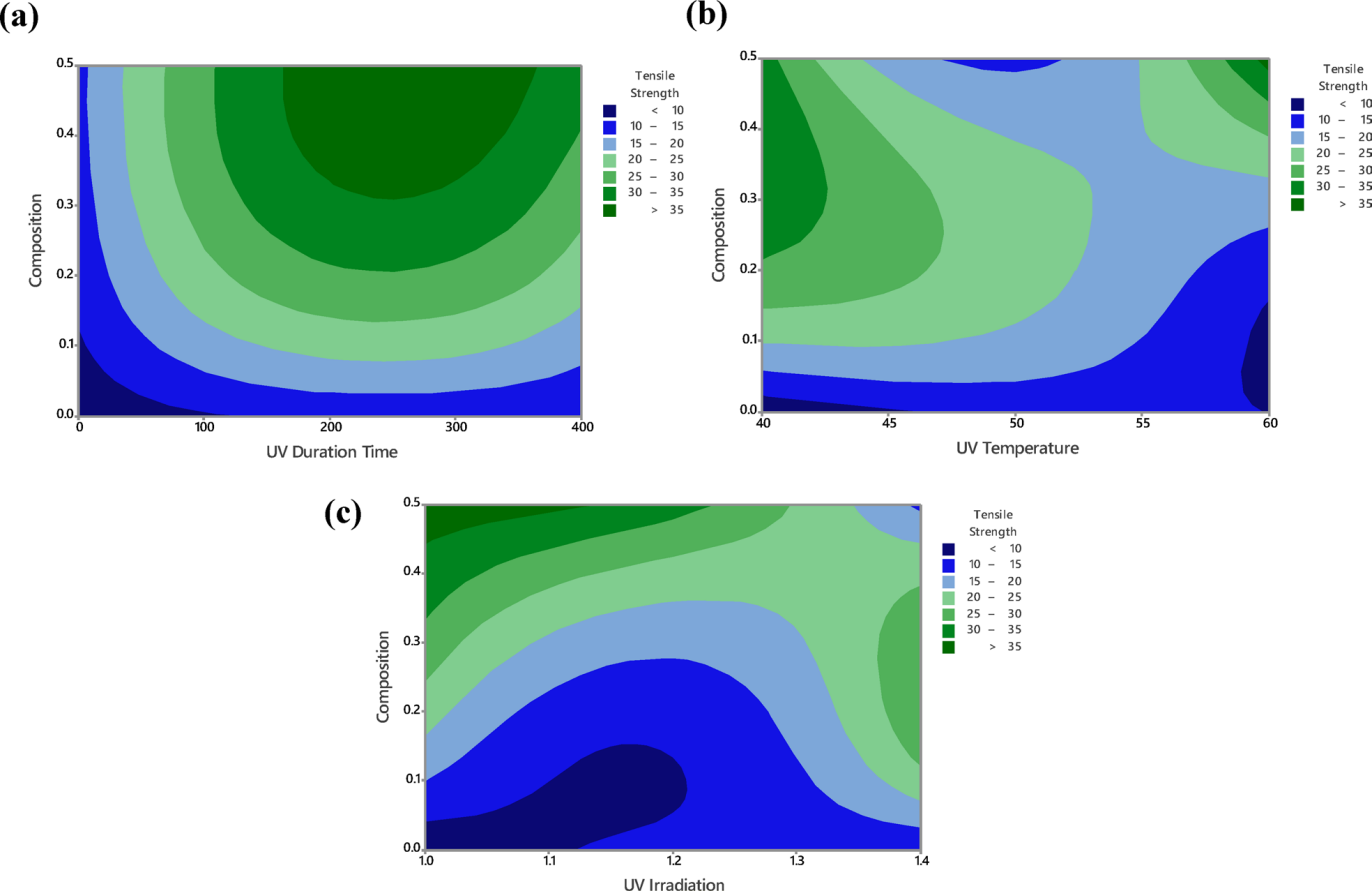
Tensile strength contour maps for (**a**) Composition against UV Duration Time, (**b**) Composition vs. UV Temperature, and (**c**) Composition vs. UV Irradiation.

The contour plots as shown in Fig. 9(a) demonstrate the combined influence of composition and UV duration time on the tensile strength of PU/ND nanocomposites. The contour plots show that tensile strength improved at 0.5 wt% PU/ND. This enhancement in tensile strength is due to the reinforcement of the polymer matrix by ND nanoparticles, which enhances its durability. The prolonged UV exposure time of 200–400 h decreases tensile strength by degrading the polymer matrix. Figure 9(b) shows how composition and UV temperature affect the tensile strength. The contour plot demonstrates that higher ND concentrations lead to substantial increases in tensile strength across all UV temperature ranges (40–60). The UV temperature changes show only small effects on tensile strength because composition is the main factor determining strength, while temperature has only minor effects on material strength. The tensile strength showed only slight variation with changes in UV temperature because composition was the primary factor determining strength, while temperature had a minor effect on mechanical performance. The combined impact of composition and UV irradiation is illustrating in Fig. 9(c). The contour plots findings revels that tensile strength improves with increasing ND concentration at 0.5 wt% PU/ND composite. The different UV irradiation levels show slight changes in tensile results, but those changes show less impact than material composition. With lower ND content, tensile strength decreases under different UV irradiation conditions. The contour plots demonstrate that composition has the strongest effect on tensile strength, whereas UV duration, temperature, and irradiation have the weakest effects. The addition of nanodiamond reinforcement dramatically enhance the structural strength of polyurethane while increasing its resistance to UV damage.

##### Young’s modulus

Young’s modulus data were examined using the Taguchi method, considering the experimental factors of composition, UV duration time, UV irradiation, and UV temperature. The results from Analysis of Variance (ANOVA) are presented in Table 11. The analysis shows that composition contributes the largest portion to the variation in Young’s modulus (40.21%), followed by UV duration time (19.13%). Interaction effects are also observed. The combination of composition and UV irradiation contributes 20.48%, while the interaction between UV duration time and UV irradiation accounts for 4.74%. All other factors contribute less than 10%. These results indicate that the composite composition has the major influence on the improvement of Young’s modulus in PU–ND nanocomposites. A regression model was employed to estimate the Young’s modulus, and the results demonstrate strong alignment with the experimental results. The coefficient of determination (R²) is 95.45%, while the adjusted R² value is 93.77%, showing that the model explains most of the variation in the response. A linear regression model has been developed to study the association between the process factors. —composition (C), UV duration time (UDT), UV irradiation (UI), and UV temperature (UT)—and Young’s modulus. The resulting predictive equation for all specimens is given in Eq. (13).

13 $$\begin{gathered}{\mathrm{Young}}^{\prime}{\mathrm{s}}\,{\text{modulus (MPa)}}=-9.3{\text{ }} + \;1452\;\left( C \right){\text{ }} + \;0.568\;\left( {UDT} \right) + \;294.6\;\left( {UI} \right) - \;3.528\;\left( {UT} \right){\text{ }}\hfill \\- \;0.081\;\left( C \right)*\left( {UDT} \right){\text{ }} - \;1136\;\left( C \right)*\left( {UI} \right){\text{ }} - \;0.489\;\left( {UDT} \right)*\left( {UI} \right)\end{gathered}$$

**Table 11:** Young’s modulus variance analysis.

Source	DF	Seq SS	Contribution	Adj SS	Adj MS	F-Value	P-Value
Composition	1	13415.3	40.21%	9005.90	9005.90	112.63	0.000
UV Duration Time	1	6384.5	19.13%	1879.08	1879.08	23.50	0.000
UV Irradiation	1	2496.9	7.48%	3396.61	3396.61	42.48	0.000
UV Temperature	1	854.2	2.56%	4838.70	4838.70	60.52	0.000
Composition*UV Duration Time	1	280.3	0.84%	42.46	42.46	0.53	0.475
Composition*UV Irradiation	1	6833.8	20.48%	8415.66	8415.66	105.25	0.000
UV Duration Time*UV Irradiation	1	1582.5	4.74%	1582.51	1582.51	19.79	0.000
Error	19	1519.2	4.55%	1519.20	79.96		
Total	26	33366.7	100.00%				

R-sq = 95.45 %  R-sq(adj) = 93.77 %

**Fig. 10:**
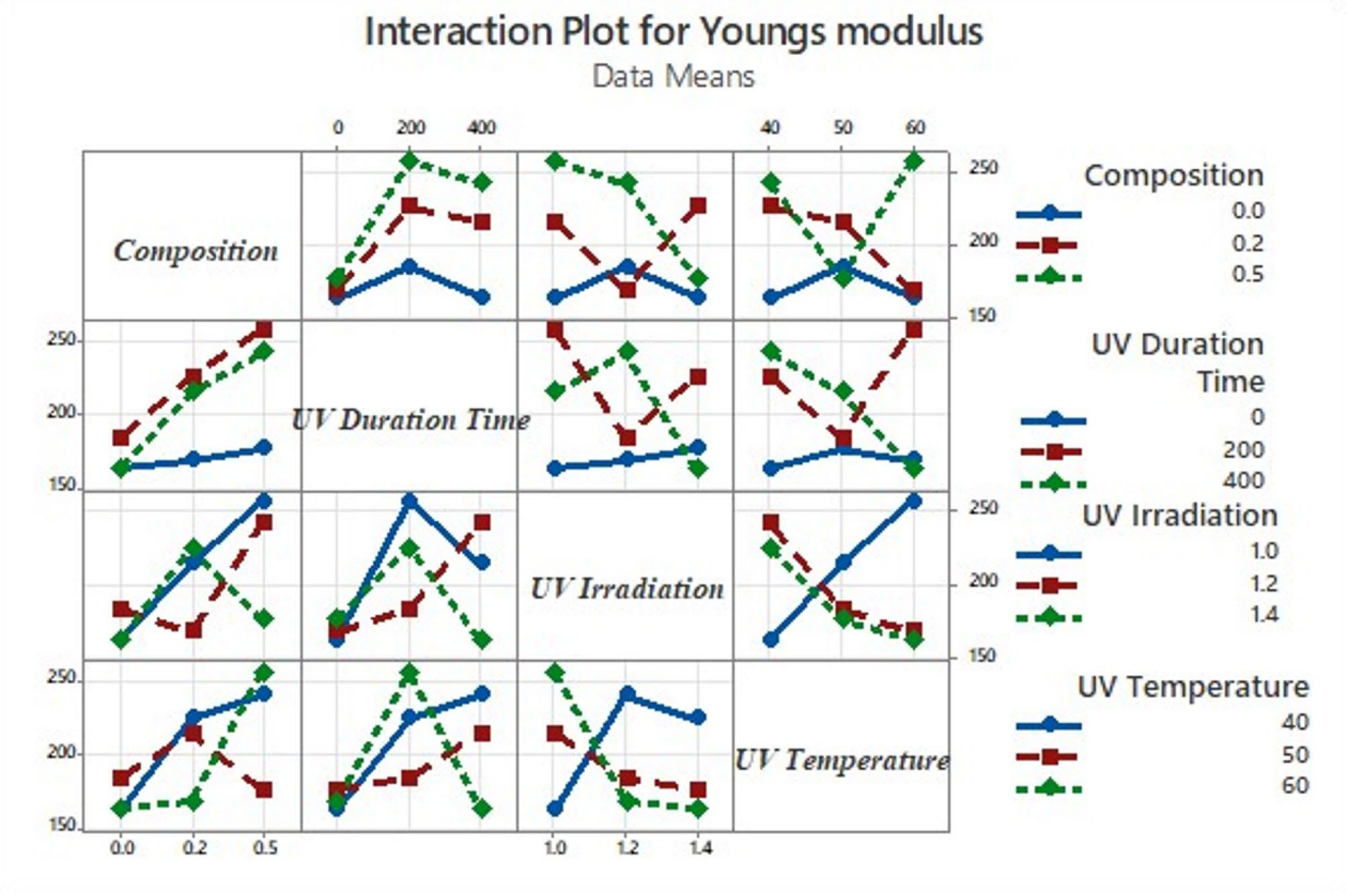
Interaction plots for Young’s modulus.

**Fig. 11:**
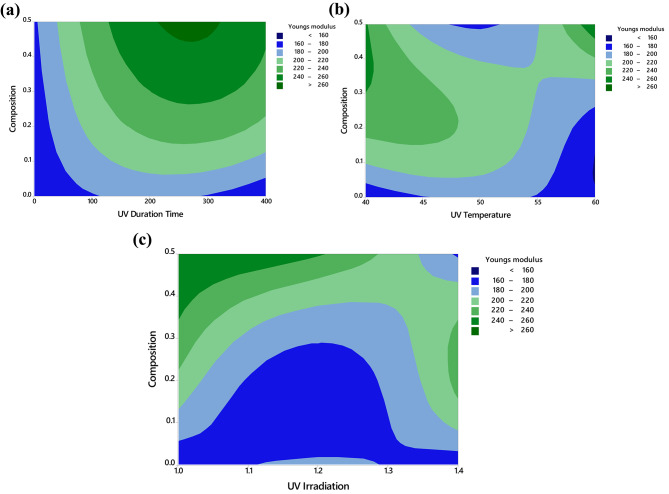
Young’s modulus contour maps for a) Composition vs UV Duration Time, b) Composition vs UV Temperature, and c) Composition vs UV Irradiation.

Figure 10 shows the interaction graphs of Young’s modulus for the polyurethane/nanodiamond (PU/ND) nanocomposite, demonstrating the integrated impact of composition and UV exposure on material stiffness. The Young’s modulus shows a continuous improvement with increasing nanodiamond loading from 0.2 to 0.5 weight per cent because nanodiamonds create a rigid structure that improves load transmission and restricts polymer chain movement. Continuous UV exposure for 200 to 400 h results in a gradual decline in modulus due to photo-oxidative deterioration via chain scission, crosslink breakage, and microstructural damage. The interaction plot shows that nanodiamond significantly enhances tensile strength but also indicates the necessity to control numerous processing factors to achieve optimal mechanical characteristics—the combined effect of composition and UV duration, as shown in Fig. 11(a). The contour maps indicate that Young’s modulus increases significantly as the nanodiamond (ND) concentration increases. The maximum stiffness values are obtained at 0.5 wt% ND, which shows that nanodiamonds provide a strong strengthening effect to the polyurethane matrix. The rises in UV exposure time from 200 to 400 leads to a moderate reduce in Young’s modulus, as UV crosslinking and oxidation increase surface hardness while reducing polymer chain mobility. The Young’s modulus decreases at a low ND concentration because exposure time had no effect on its value, which shows that the composition factor is the primary element that determines stiffness. Figure 11(b) shows that composition has a greater effect on Young’s modulus than UV temperature. Increasing the 0.5 PU/ND loading consistently results in higher stiffness across all UV temperatures (40–60). UV-induced temperature changes cause only small variations in modulus because temperature primarily drives ageing processes, which require proper reinforcement to induce stiffness changes. In Fig. 11(c), the graph demonstrates that Young’s modulus shows a considerable increase across all irradiation levels as ND concentration increases. The lower ND concentration and lower UV irradiation range result in the lowest measured stiffness values. The UV irradiation increase results in a modulus increase, which shows a minor effect. The highest modulus measurements occur at 0.5 wt% PU/ND because nanodiamond reinforcement effectively enhances material stiffness. Therefore, the contour plots show that Young’s modulus depends mostly on composition, while UV duration, UV temperature, and UV irradiation have minimal effects. The addition of nanodiamonds leads to significant improvements in stiffness and preserves mechanical integrity under prolonged UV exposure, as the material exhibits greater resistance to UV-induced damage.

##### Hardness

The hardness of the composites was analysed using the Taguchi method, with composition, UV Duration Time, UV Irradiation, and UV Temperature as the experimental parameters. According to the “larger is better” criterion, the analysis result. As shown in Table 12, the UV Duration Time is the most significant factor, contributing 49.60% to hardness, followed by composition (42.56%), UV Irradiation (0.47%), and UV Duration Time*UV Temperature (2.81%). Whereas other factors contributed to less than 10%. The regression model used to predict the hardness of PU-ND composites is excellent precision, with a coefficient of determination (R^2^) is 97.08% while the adjusted R^2^ value is 96.01%. These data validate the strength and accuracy of the models in explaining the hardness characteristics. A linear regression model is developed to investigate the interaction between the process parameters of composition(C), UV Duration time(UDT), UV Irradiation (UI), and UV Temperature (UT) and the Hardness. The forecasting regression equation for PU-ND composites is present in Eq. (14).

14 $$\begin{gathered}{\mathrm{Hardness(HV)}} =56.26{\text{ }} + \;124.8\;\left( C \right){\text{ }} + \;0.0708\;\left( {UDT} \right) + \;7.22\;\left( {UI} \right) + \;0.138\;\left( {UT} \right) \hfill \\- 0.0493\;\left( C \right)*\left( {UDT} \right)- \;74.7\;\left( C \right)*\left( {UI} \right) - \;0.002276\;\left( {UDT} \right)*\left( {UT} \right)\end{gathered}$$

Regression equation for Hardness.

**Table 12:** Hardness variance analysis

Source	DF	Seq SS	Contribution	Adj SS	Adj MS	F-Value	P-Value
Composition	1	1297.97	42.56%	56.702	56.702	12.11	0.003
UV Duration Time	1	1512.50	49.60%	24.024	24.024	5.13	0.035
UV Irradiation	1	14.22	0.47%	8.112	8.112	1.73	0.204
UV Temperature	1	1.39	0.05%	7.095	7.095	1.52	0.233
Composition*UV Duration Time	1	44.08	1.45%	10.004	10.004	2.14	0.160
Composition*UV Irradiation	1	4.69	0.15%	36.344	36.344	7.76	0.012
UV Duration Time*UV Temperature	1	85.61	2.81%	85.610	85.610	18.29	0.000
Error	19	88.94	2.92%	88.940	4.681		
Total	26	3049.41	100.00%				

R-sq = 97.08 %   R-sq(adj) = 96.01 %

**Fig. 12:**
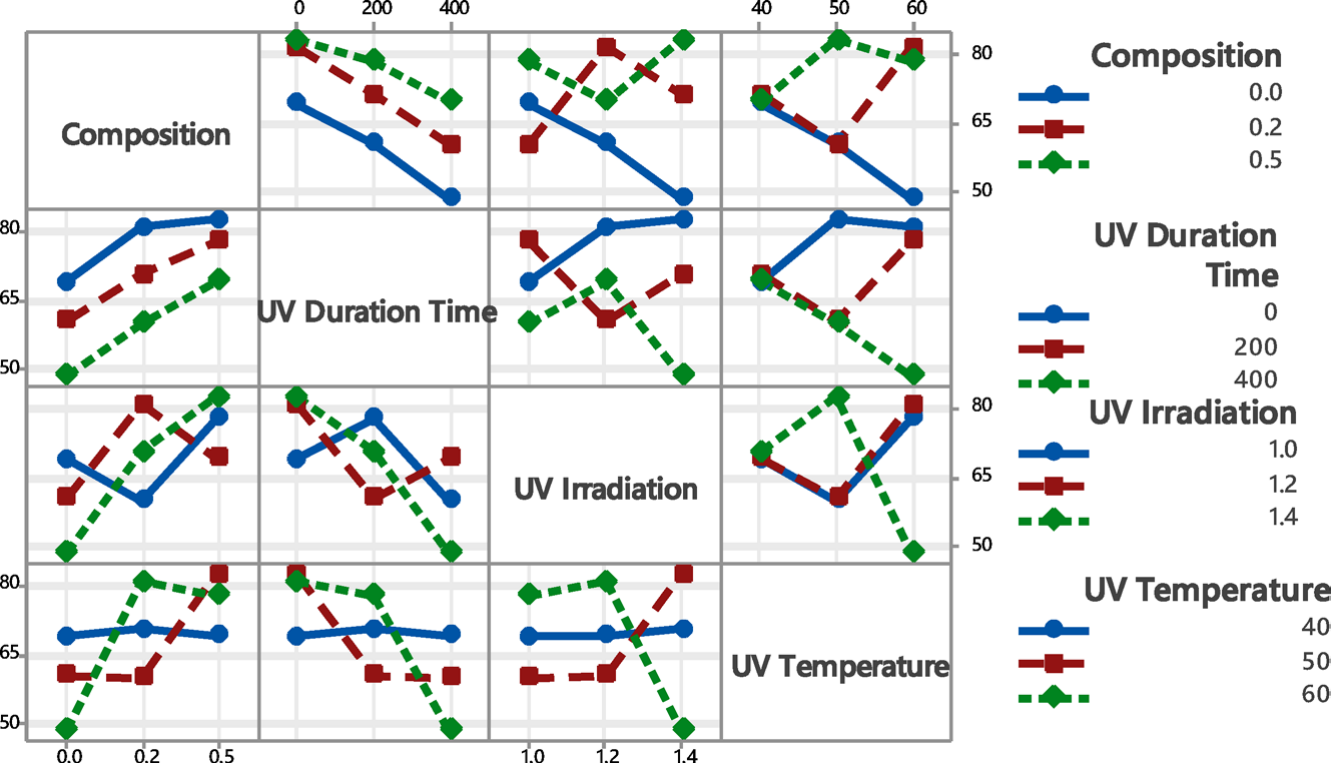
Interaction plots for Hardness.

**Fig. 13:**
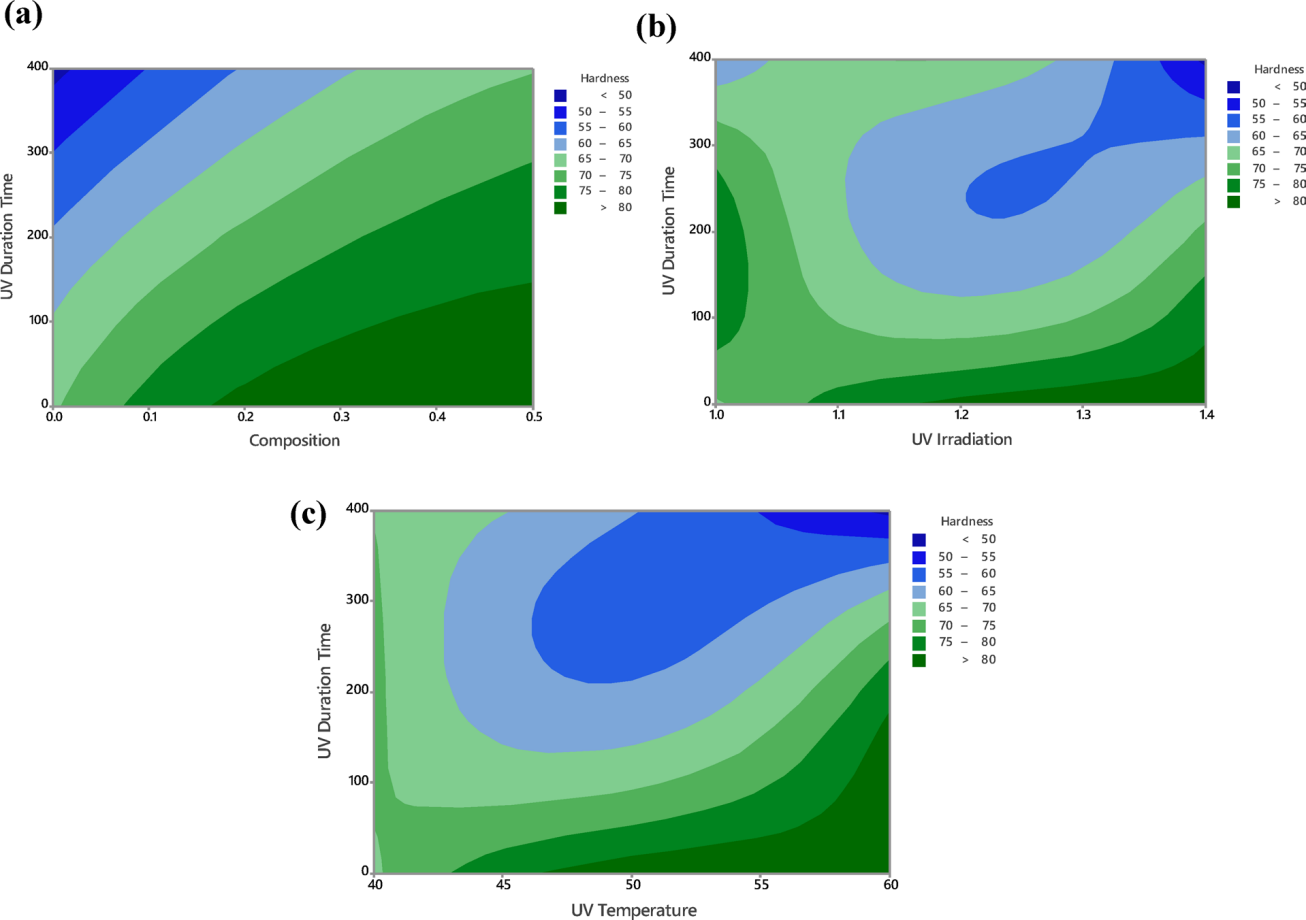
Hardness for (**a**) UV Duration time vs. composition (**b**) UV Duration time vs. UV Irradiation (**c**) UV Duration time vs. UV Temperature.

Figure 12 shows the interaction effects of composition, UV Duration Time, UV Irradiation, and UV Temperature on hardness. The interaction plot demonstrate that UV Duration Time and composite material are the most dominant factors impacting hardness, while the, UV Irradiation, and UV Temperature, are small impact on hardness. Figure 13(a) shows the combined impact of UV exposure duration time and composition on the hardness of PU/ND nanocomposites. The results show that increasing ND content leads to significant hardness improvements while the highest hardness value observed at 0.5 wt% PU/ND demonstrating nanodiamonds act as strong reinforcement effect. The hardness gradual reduction in during UV duration time between 0 and 400 h because low ND concentration leads to polymer breakdown and chain scission and surface embrittlement. However, higher ND loading was found to sustain hardness at prolonged exposure durations implying the importance of the composition. The Fig. 13(b) shows the shorter UV Duration intervals (< 200), higher irradiation levels (> 50) shows higher hardness values, due to surface hardening effects. The hardness decreases at all UV irradiation levels when UV Duration time increases (200–400) indicating UV light causes gradual material degradation. Although irradiation effects on hardness is less substantial than the effects of material composition. Figure 13(c) the contour map shows how UV Duration time and UV temperature, affect the material’s hardness. The result show that hardness increases with higher UV temperature levels during shorter UV Duration times (< 200) because UV light creates surface hardening through crosslinking and oxidation. The hardness of all UV duration time shows a continuous decrease because the polymer matrix undergoes more photo degradation as prolonged UV Duration time (200–400), this reduction reflects development of chain scission and microstructural degradation and surface degeneration. The results shown that 0.5 PU/ND reinforcement has a significant increase hardness. UV duration time has a primary impact, lowering hardness with longer UV exposure time. Whereas UV irradiation and UV temperature have comparably minor effects. In summary, adding nanodiamonds improves surface hardness and contributes to the mechanical stability of polyurethane under UV aging conditions.

### Machine learning

#### Tensile strength

**Fig. 14:**
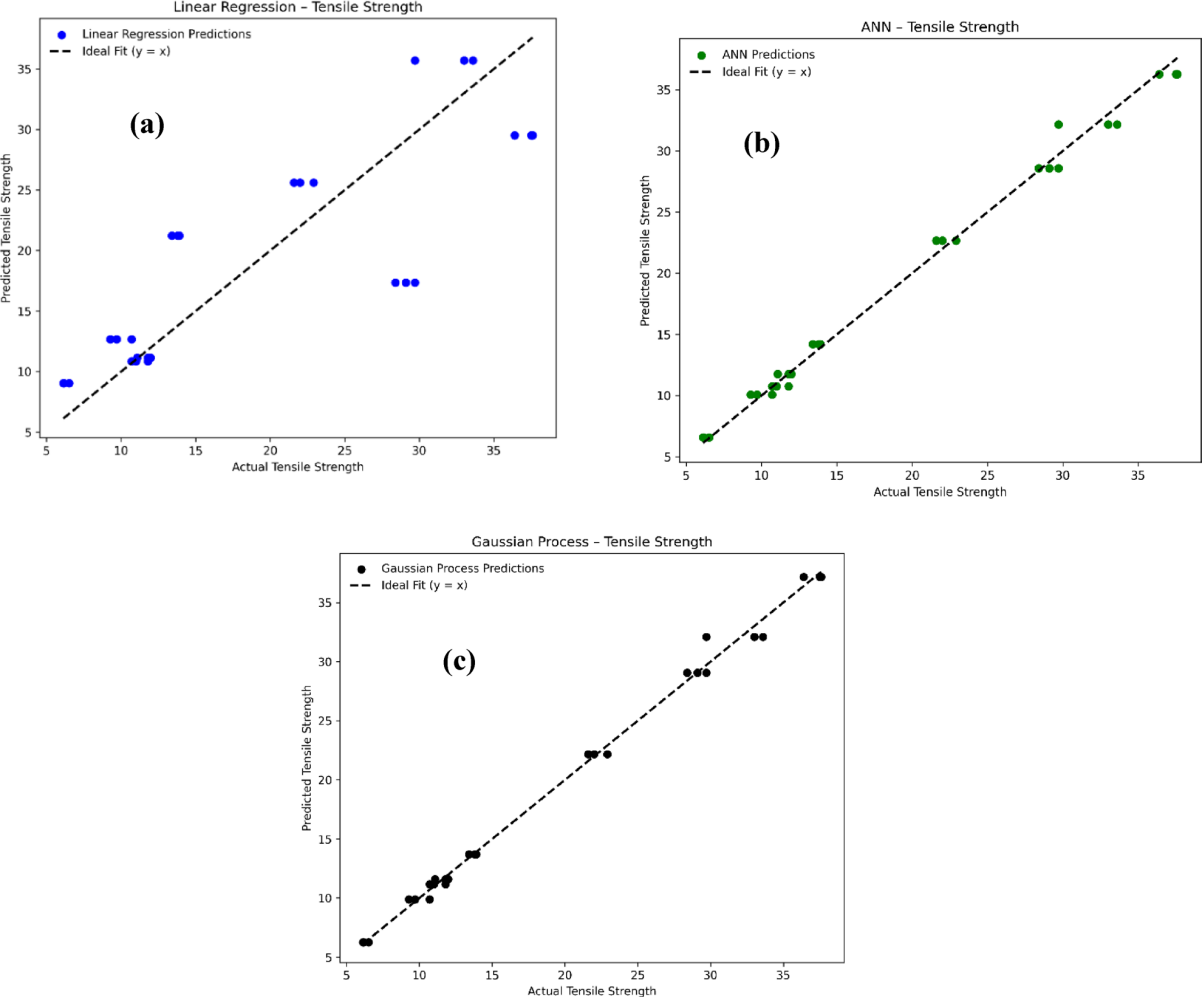
Actual vs. Predicted values for tensile strength of (**a**) linear regression (**b**) ANN (**c**) Gaussian process.

Figure 14a–c compares measured and predicted tensile strength values obtained from three models: Linear Regression, Artificial Neural Network, and Gaussian Process Regression. In each graph, the measured values are represented on the x-axis while the expected values on the y-axis. The blue points indicate individual specimens, whereas the black diagonal line depicts the ideal case where predicted outcomes and measurement are identical (y = x). Figure 14a presents the results from the linear regression model. The technique achieves an R² value of 0.70, with MSE, RMSE, MAE, and MAPE values are 33.11, 5.75, 4.49, and 0.24. Figure 14b shows the prediction results from the ANN model. This predictive model gives an R² of 0.99, with MSE, RMSE, MAE, and MAPE of 0.71, 0.84, 0.65, and 0.03, respectively. Figure 14c depicts the predictive accuracy of the Gaussian process model. The predictive model produces the R² of 0.99, along with MSE, RMSE, MAE, and MAPE of 0.51, 0.71, 0.52, and 0.02, respectively. The comparison of these results shows that the Gaussian process model yields the closest match between predicted and measured tensile strength, followed by the ANN model, whereas the linear regression model exhibits larger prediction errors.

**Fig. 15:**
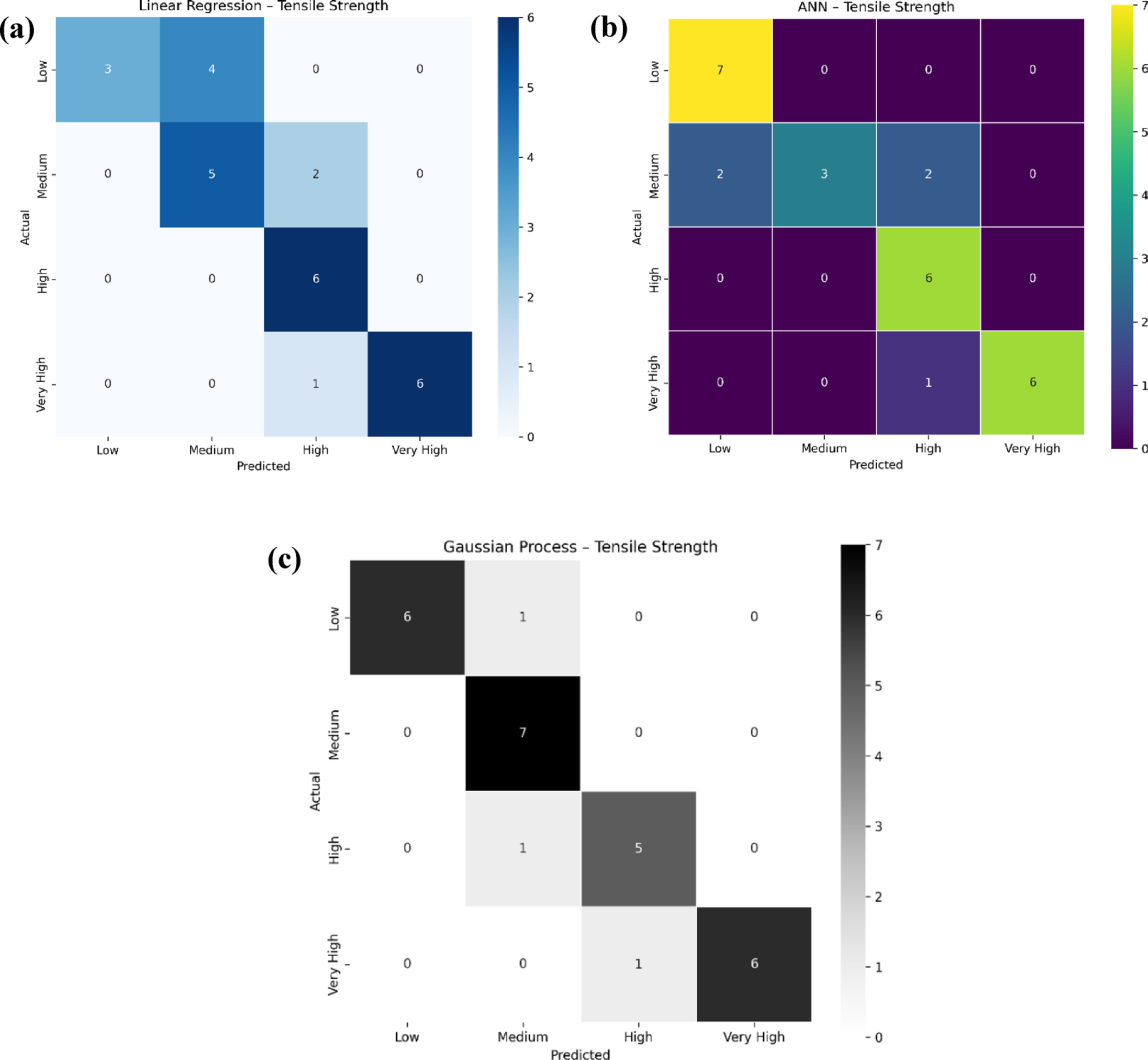
Confusion matrices for tensile strength with (**a**) linear regression (**b**) ANN (**c**) Gaussian process.

The confusion matrixes of the linear regression, ANN, Gaussian categorization models as present in Fig. 15a-c. The tensile strength classified in to Four class: low, medium, high, and very high. The confusion matrices show the distribution of classes across their entire distribution. The categorization system uses percentages which include 0th 25th 50th 75th and 100th percentages. The ranges of values from 0% to 25% percentage was classified as low, from 25% to 50% percentage as medium, from 50% to 75% percentage as high, and above 75% percentage as very high. The statistical basis for quantile-based binning supports its usage to analyze categorizing outcomes using confusion matrices. The true labels appear in the rows of the matrix while the estimated labels display their values in the columns. The confusion matrices presented in Fig. 15(a) demonstrate that the linear regression model successfully predicts tensile strength classifications. The system correctly identifies 3 of 7 low categories while 4 are incorrectly identified as medium. The system correctly predicts 5 of 7 medium tensile strength samples but 2 samples were incorrectly identified as high. The high classification 6/6 are accurately predicted.in the very high categories 6 of 7 are classified as correctly with 1 incorrectly classified as high. These findings demonstrate that the model reliable and robust predictive accuracy. Figure 15(b) present the ANN model confusion matrices for identifying tensile strength. Low and high tensile strength categories had 7of 7, and 6 of 6 are accurately predicted. The medium classification 3of 7 are accurately categorized other 2 are incorrectly classified as low and high. the very high tensile strength category 6 of 7 are precisely classified and 1 are incorrectly classified as high. In summary, the ANN model prediction is best suited for predicted for lower and higher tensile strengths. Figure 15(c) illustrate the confusion matrices of the Gaussian model shows the categorizations of tensile strength .in the low tensile strength category 6 of 7 are precisely classified and 1are incorrectly classified as medium. The medium category 7 of 7 are precisely predicted. The high category 5of 6 are precisely classified and 1 are incorrectly categorized as medium. These findings demonstrate the model accuracy and predictive ability.

**Fig. 16:**
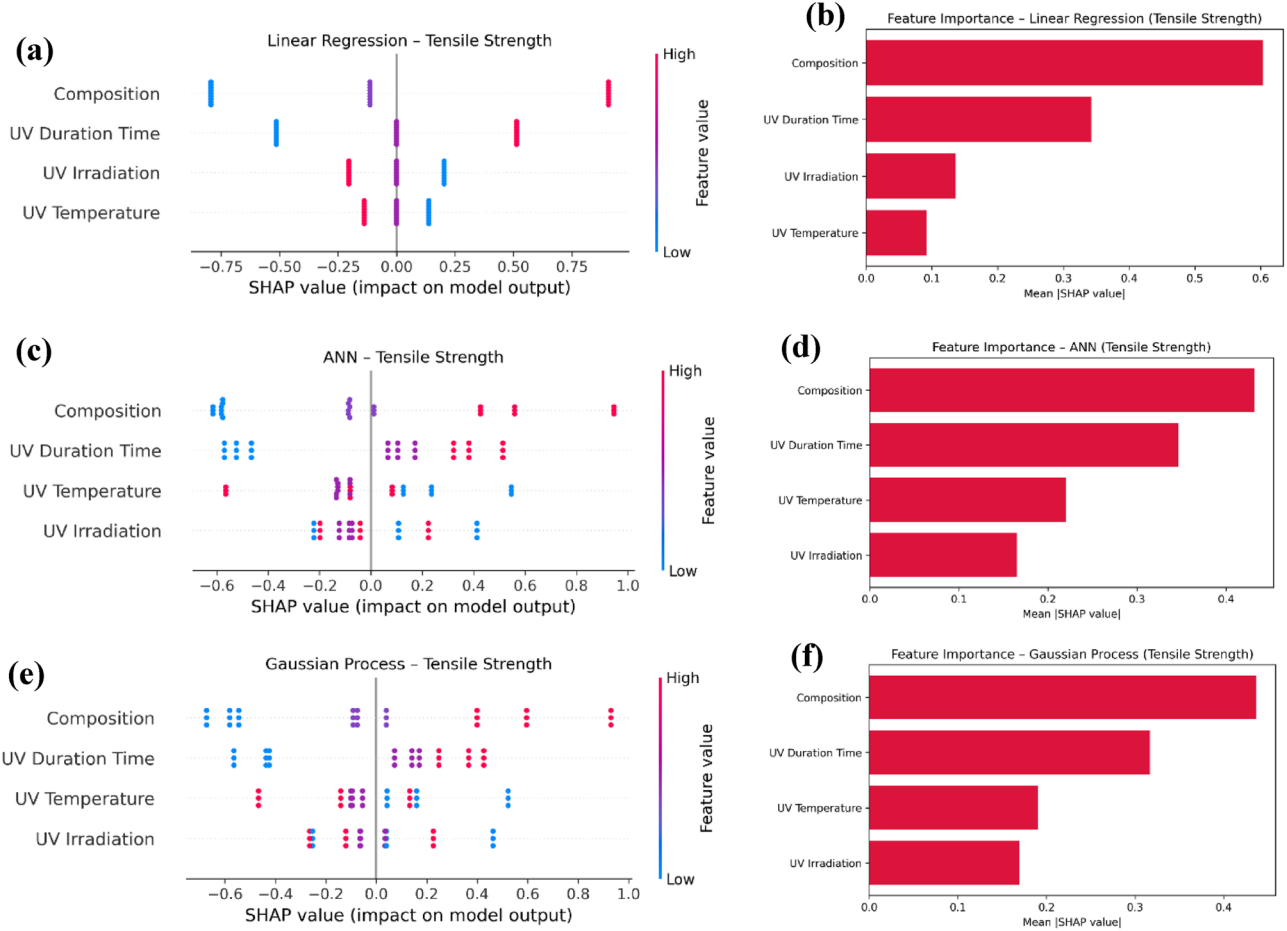
SHAP analysis & Feature importance evaluation for tensile strength of **a**), **b**) Linear regression, **c**),** d**) ANN, **e**), **f**) Gaussian process.

The SHAP analysis for tensile strength prediction used three modeling techniques: linear regression, ANN, and Gaussian process models which are illustrated in Figure 16a-f. These predictive models emphasize the feature importance and its distribution of SHAP values. In the linear regression predictive model Figure 16a-b demonstrate that the composition (wt%) and UV Duration time are the two most important affected parameters which display SHAP values which range from -0.75 to +0.75 and -0.50 to +0.50 and their feature importance values which are 0.6 and 0.35. As composition increases, tensile strength increases, whereas prolonged UV exposure (200–400 h) decreases tensile strength by causing polymer matrix degradation. UV Irradiation and UV temperature has a moderate influence on predicting tensile strength. In the ANN model (Figure 16c-d), the identified composition (wt%) is the major influencing factor with the SHAP value ranges from -0.6 to +1.0 with and Mean shape value of 0.45, whereas UV duration time of 0.35, UV temperature of 0.25, and UV Irradiation of 0.15. In Figure 16e-f, the Gaussian process reveals that composition (wt%) has SHAP values ranging from -0.65 to +1.0 with a mean SHAP value of 0.43, while UV Duration time is 0.32, and UV Temperature and UV Irradiation play a minor role. Therefore, the data indicated that composition and UV duration period are the highly dominant impacts on tensile strength, followed by UV temperature and UV Irradiation. The SHAP analysis demonstrates that ANN and Gaussian models can effectively predict the tensile strength because they successfully capture nonlinear interactions compared to linear regression.

#### Young’s modulus

**Fig. 17:**
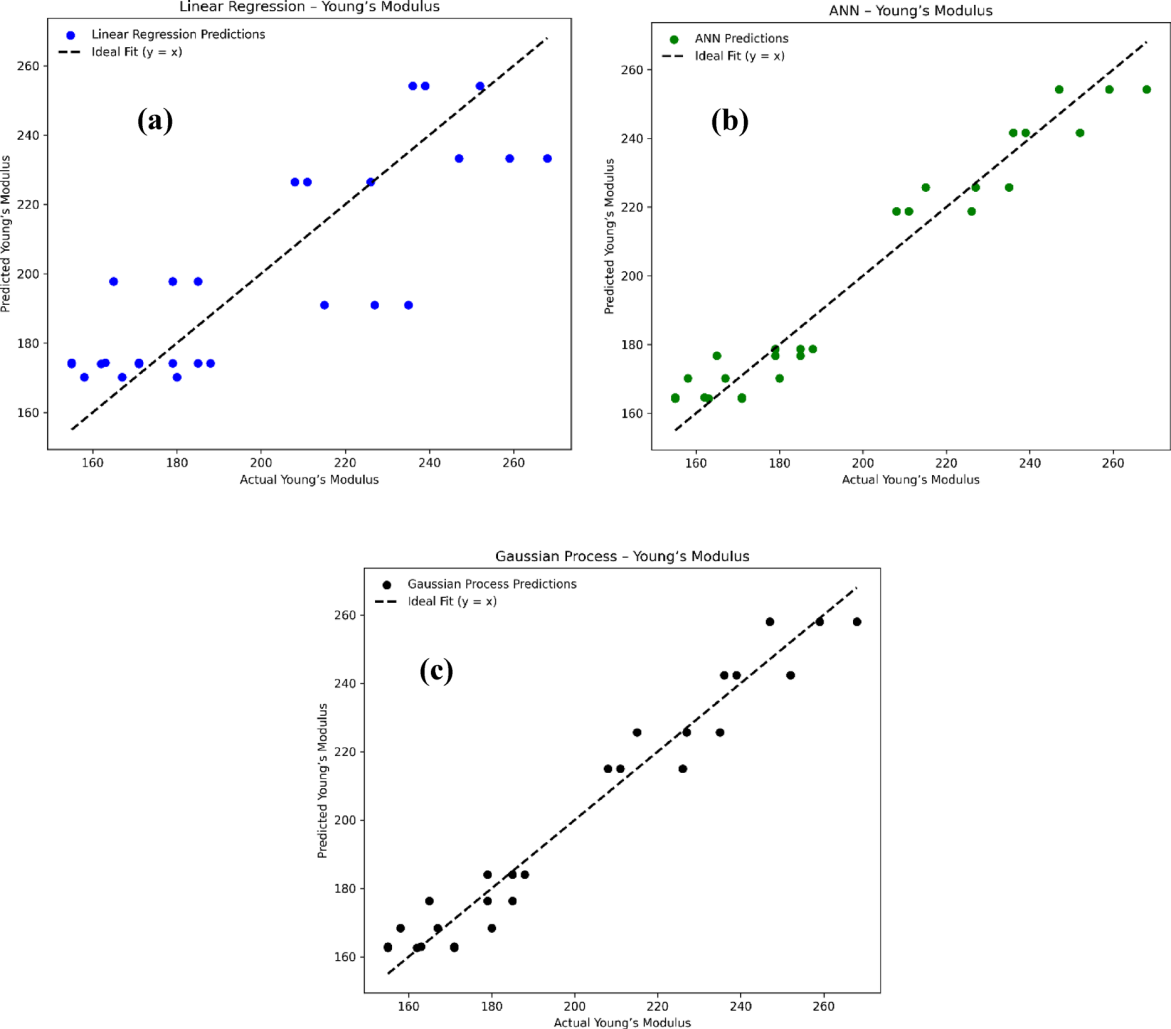
Actual vs. Predicted values of Young’s modulus of (**a**) linear regression, (**b**) ANN, (**c**) Gaussian process.

Figure 17a–c shows the comparison between measured Young’s modulus values and predicted Young’s modulus values derived from three models which include Linear Regression Artificial Neural Network and Gaussian Process Regression. The graphs display actual values on the x-axis and estimated values on the y-axis, allowing direct comparison between the experimental results and the model outputs. Figure 17a presents the results from the linear regression model. The predictive model yields an R-square value of 0.69, having MSE, RMSE, MAE, and MAPE of 378.36, 19.45, 16.11, and 0.08, respectively. Figure 17b shows the prediction results from the ANN model. This model yields an R² of 0.94, having MSE, RMSE, MAE, and MAPE of 63.28, 7.95, 7.06, and 0.03, respectively. Figure 17c demonstrate that the results obtained from the Gaussian process model for Young’s modulus. The model produces an R² of 0.95, with MSE, RMSE, MAE, and MAPE of 55.90, 7.47, 6.41, and 0.03, respectively. A comparison of these results shows that the Gaussian process model provides the closest match between estimated and measured values of Young’s modulus, followed by the ANN model, while the linear regression model shows larger prediction errors.

**Fig. 18:**
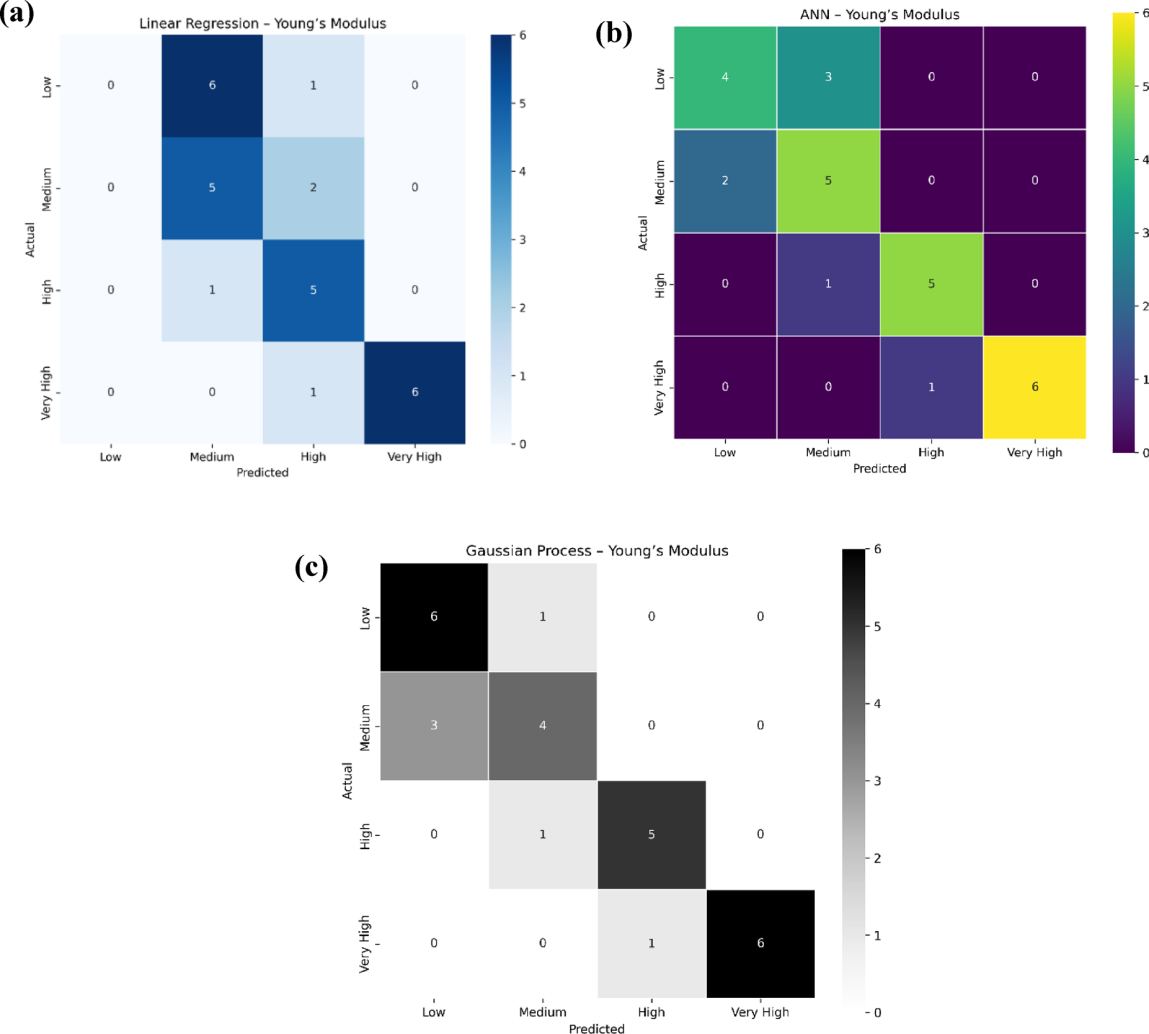
Confusion matrices of Young’s modulus with (**a**) linear regression, (**b**) ANN, and (**c**) Gaussian process.

Figure 18a presents the performance of linear regression in classifying the Young’s modulus into four categories: low, medium, high, and very high. In the low classification, 6 of 7 are precisely predicted, and 1 is incorrectly identified as high. In the medium categorization, 5 of 7 are precisely classified, and 2 are incorrectly classified as high. In the high categorization, 5 of 6 are perfectly predicted, and 1 is incorrectly determined as medium. In the very high category, 6 of 7 are exactly classified, and 1 is incorrectly identified as high. Therefore, the model more accurately predicted the extreme value of the Young modulus. Figure 18(b) presents the confusion matrices for the performance of the ANN model in categorising Young’s modulus. In the low classification, 4 of 7 are correctly predicted, and 3 are incorrectly identified as medium. In the medium classification, 5 of 7 are precisely identified, and 2 are incorrectly classified as low. In the high classification, 5 of 6 are correctly classified, while 1 is incorrectly classified as medium. In the very high classification, 6 of 7 were correctly classified 1 was incorrectly classified as high. The findings demonstrate that, indeed, the model is reliable and robust in terms of its predictive accuracy —Figure 18(c) confusion matrices for the Gaussian process performed in categorizing Young’s modulus. In the low classification, 6 of 7 are precisely predicted. At the same time, 1 is incorrectly predicted as medium. In the medium classification, 4 of 7 are precisely identified, and 3 are incorrectly identified as low. In the high classification, 5 of 6 are exactly predicted. In contrast, 1 is incorrectly determined as medium. In the very high classification, 6 of 7 are accurately identified, and 1 is incorrectly classified as high. In summary, the confusion matrices demonstrate the robust and predictive accuracy of the Gaussian process.

**Fig. 19:**
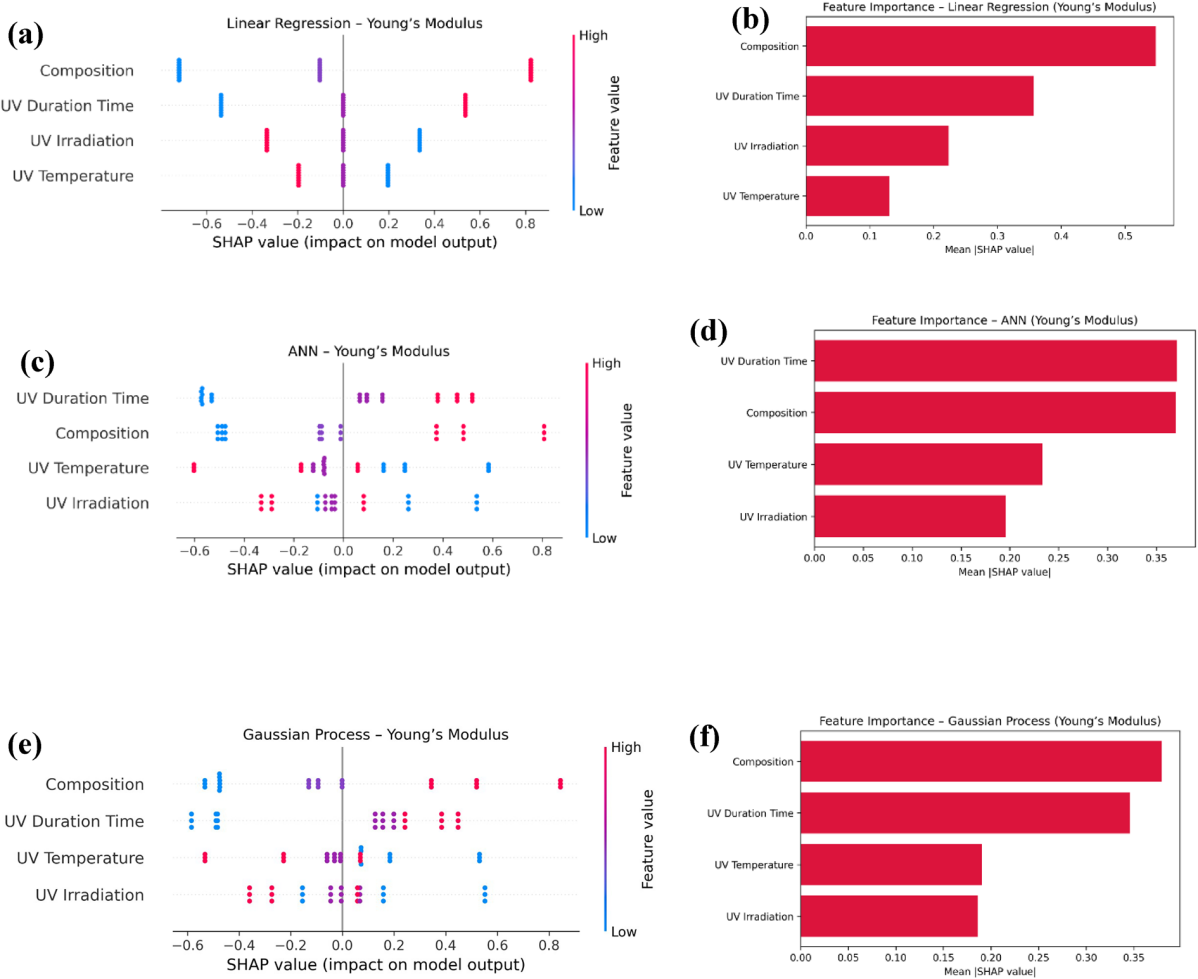
SHAP analysis and Feature importance for Young’s modulus of** a**), **b**) Linear regression, **c**), **d**) ANN, **e**), **f**) Gaussian process.

The SHAP examination for the prediction of the Young’s modulus shows results from three methods which include linear regression and ANN and Gaussian Process, as presented in Fig. 19 (a-f). as well as the feature importance and SHAP value distribution for the linear regression model as shown in Fig. 19a-b composition shows the most important feature with a range of -0.6 to + 0.8 SHAP values. It has been supported by the feature importance plots, with the Mean shap value of 0.56, whereas UV Duration Time of 0.35, UV Irradiation of 0.23, and UV Temperature of 0.13. ANN model in the Fig. 19c-d UV Duration time and composition are the most strongly significant impacted parameters with a SHAP value of -0.6 to + 0.8 and − 0.6 to + 0.6 and its respective feature importance values of 0.39 and 0.39. At the same time, UV Irradiation and UV Temperature have a minor impact on the prediction of Young’s modulus. In the Gaussian Process Fig. 19e-f composition, demonstrating the strongest SHAP value of -0.6 to + 0.8 and the largest mean SHAP value of 0.39, showing that it is the most significant nonlinear effect on Young’s modulus and UV Duration time, while UV Irradiation has a moderate effect, and UV Temperature has a minor impact on Young’s modulus. In summary, composition is the most influential parameter for Young’s modulus, followed by UV Duration Time, UV temperature and UV Irradiation.

#### Hardness

**Fig. 20:**
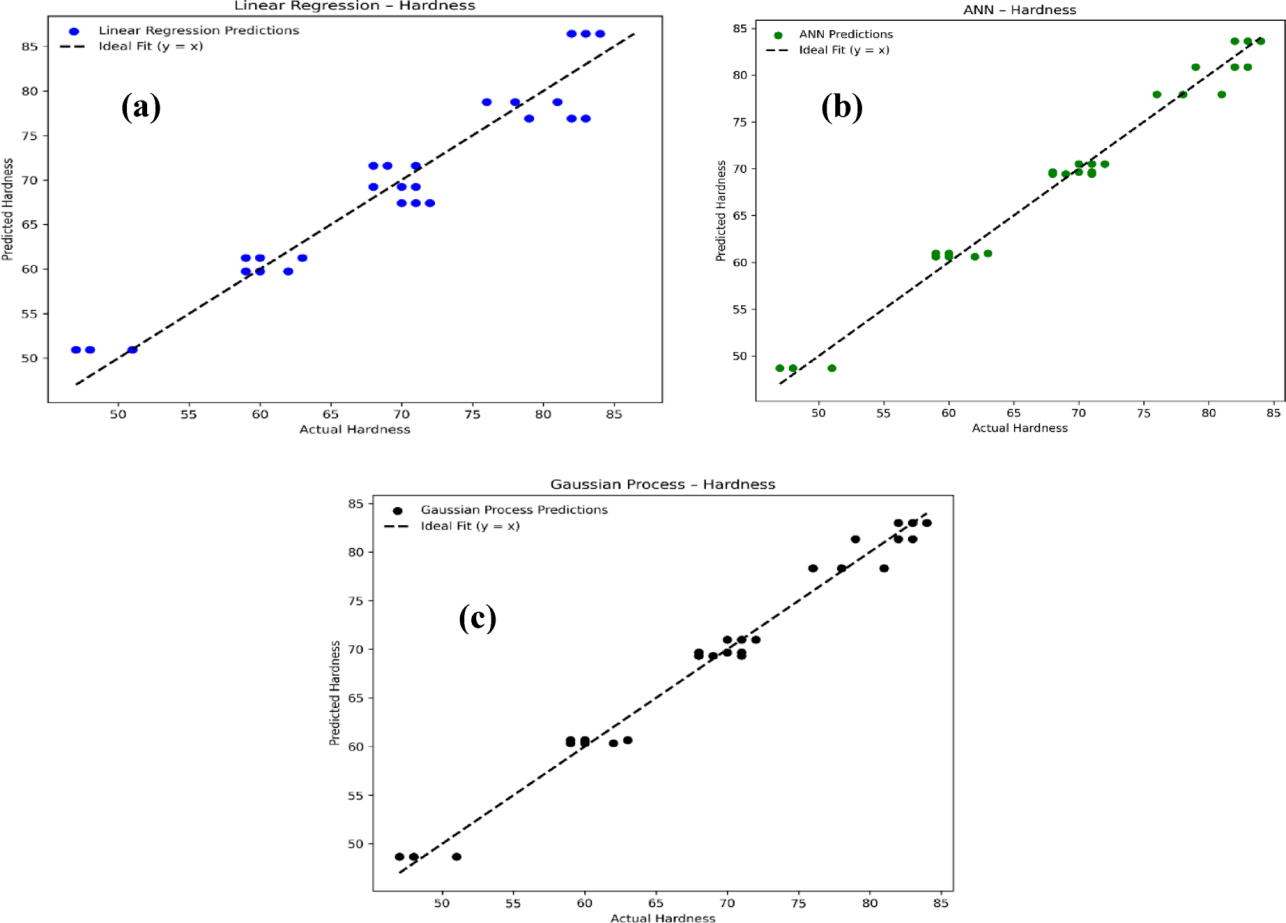
Actual and Predicted values for Hardness during (**a**) linear regression, (**b**) ANN, (**c**) Gaussian process.

**Fig. 21:**
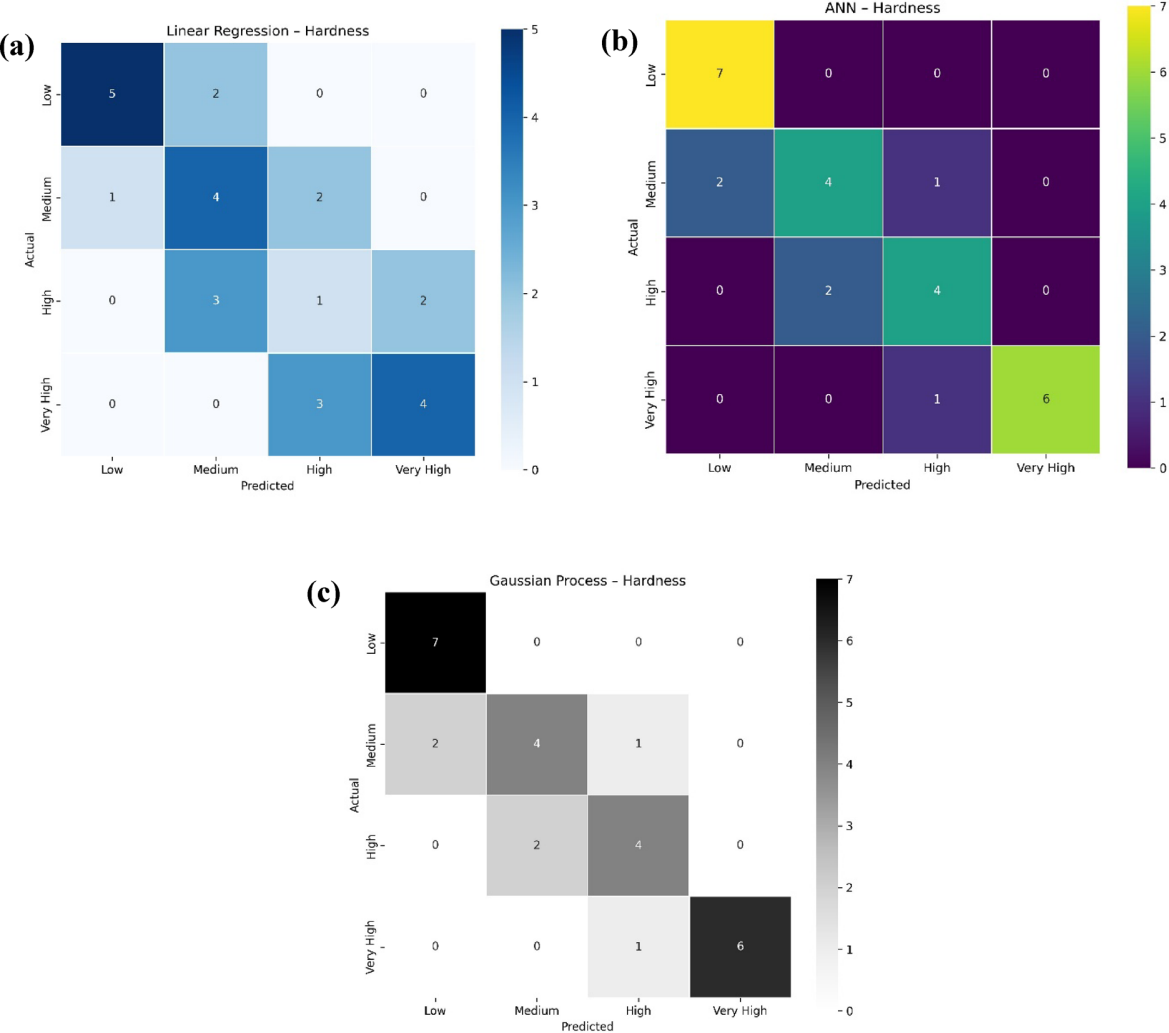
Confusion matrices for Hardness with (**a**) linear regression, (**b**) ANN, (**c**) Gaussian process.

Figure 20a–c compares the measured and predicted hardness values obtained from three models: Linear Regression, Artificial Neural Network, and Gaussian Process Regression. In each graph, the measured hardness values are placed on the x-axis, while the predicted values are shown on the y-axis. Figure 20a presents the results from the linear regression model for hardness prediction. The prediction model yields an R-square value of 0.92, including MSE, RMSE, MAE, and MAPE of 8.27, 2.87, 2.44, and 0.03, respectively. Figure 20b shows the results from the ANN model. This model produces an R² of 0.98, including MSE, RMSE, MAE, and MAPE of 2.23, 1.49, 1.30, and 0.01, respectively. Figure 20c shows the results obtained from the Gaussian process model. The model gives an R-square value of 0.98, including MSE, RMSE, MAE, and MAPE values of 2.09, 1.44, 1.23, and 0.01, respectively. These results revels that the Gaussian process model has a relative higher prediction precision compared to the ANN model, whereas the linear regression model exhibits larger prediction errors. Figure 21a shows the hardness classification results from the linear regression model, presented as a confusion matrix. In this matrix, the predicted classes appear on the horizontal axis, whereas the real classes appear on the vertical axis. In the low-hardness group, 5 of 7 samples are correctly identified, while 2 are misclassified as medium. In the medium group, 4 of 7 samples are correctly predicted, while 1 and 2 are misclassified as low and high, respectively. In the high group, 1 of 6 samples is precisely predicted, while 3 and 2 are misclassified as very high and medium, respectively. In the very high group, 4 out of 7 samples are correctly predicted, and 3 are misclassified as high. Figure 21b shows a confusion matrix to represent the ANN model’s hardness cauterization. In the low group, all 7 samples are correctly predicted. In the medium group, 4 of 7 samples are correctly classified, while 2 and 1 are incorretlypredicted as high and low, respectively. In the high group, 4 out of 6 samples are precisely predicted, whereas 2 are misclassified as medium. In the very high group, 6 out of 7 samples are correctly predicted, and 1 is misclassified as high. These results show that the ANN model provides reliable hardness classification. Figure 21c shows the confusion matrix obtained from the Gaussian process model. In the low group, all 7 samples are correctly predicted. In the medium group, 4 of 7 samples are correctly identified, while the remaining 2 and 1 are misclassified as low and high. In the high group, 4 out of 6 samples are correctly predicted, and 2 are misclassified as medium. In the very high group, 6 out of 7 samples are correctly identified, while 1 is misclassified as high. These results suggest that the Gaussian process model provides an accurate prediction aa well as classification of hardness values.

**Fig. 22:**
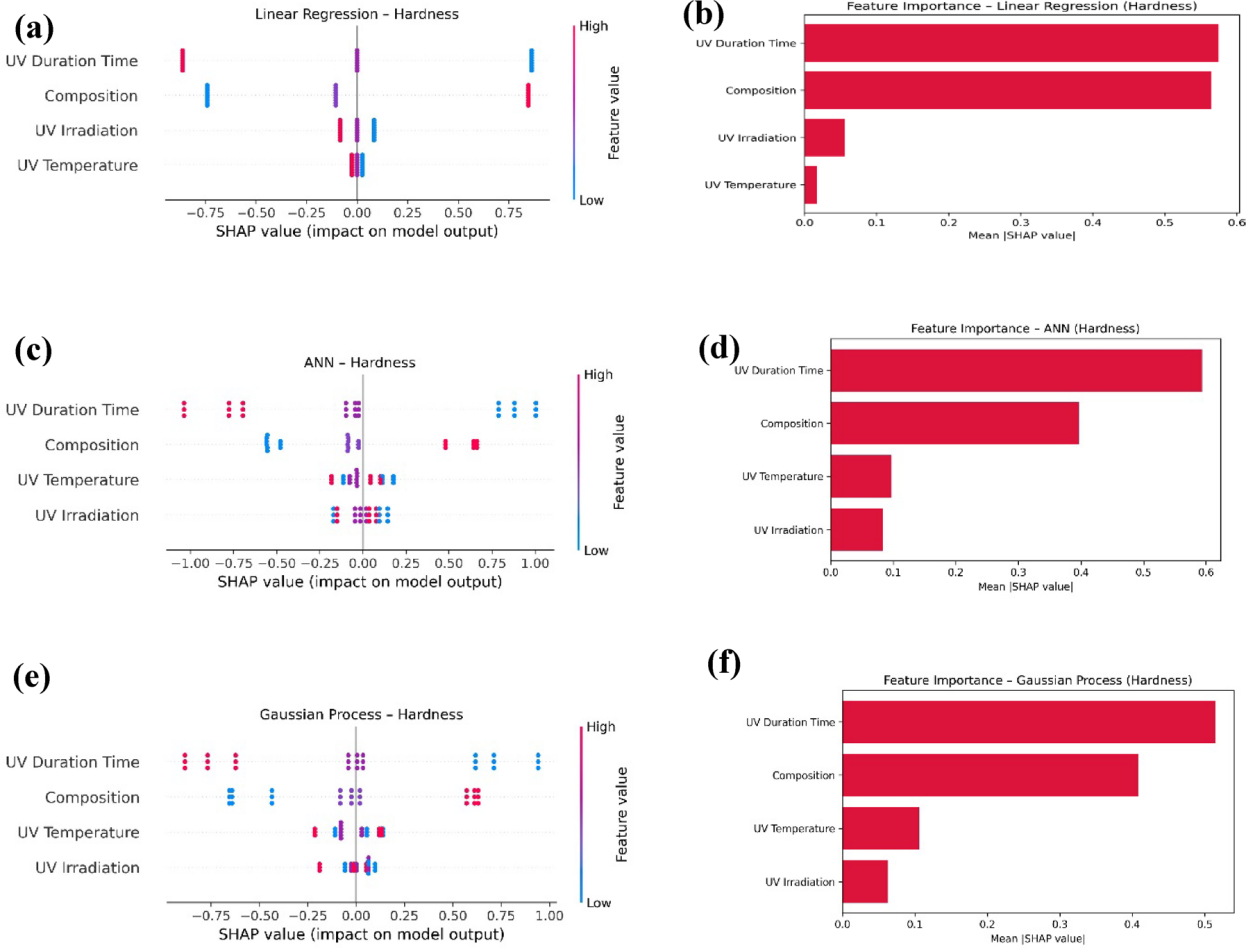
SHAP analysis and Feature importance for Hardness during **a**), **b**) Linear regression, **c**), **d**) ANN, **e**), **f**) Gaussian process.

The SHAP examination for the estimation of tensile strength using linear regression, ANN, and Gaussian process simulation, as present in Fig. 22a-f, highlights the feature importance and distribution of SHAP metric values. Using the linear regression model, Fig. 22(a-b), UV duration time and composition (wt%) the highest essential characteristics with SHAP values lying between − 0.75 and + 0.75 and their relevant feature importance plots exhibit values of 0.57 and 0.55, rises UV exposure time reduces hardness, however, increasing the 0.5 PU/ND composition increases hardness. In contrast, UV temperature and UV Irradiation have minimal contribution to predicting hardness. In the ANN model, Fig. 22(c-d) demonstrates that the UV duration time receives the highest influence with a widespread range of SHAP values around − 1.0 to + 1.0, the optimum, average SHAP value of 0.60, followed by composition (0.4), UV Temperature (0.1), and UV Irradiation with a lower contribution. In Fig. 22(e-f), the Gaussian process UV Duration time is the most prominent SHAP value of -0.75 to + 1.0, with average SHAP value of 0.5, validating that it has the highly influential quadratic effect on Hardness and composition 0.41, while UV temperature and UV Irradiation have minimal contribution to predict hardness. It is summarized that UV Duration period is the most influential parameter for Hardness, subsequently composition, UV Irradiation, and UV temperature.

#### Pearson correlation matrix

**Fig. 23:**
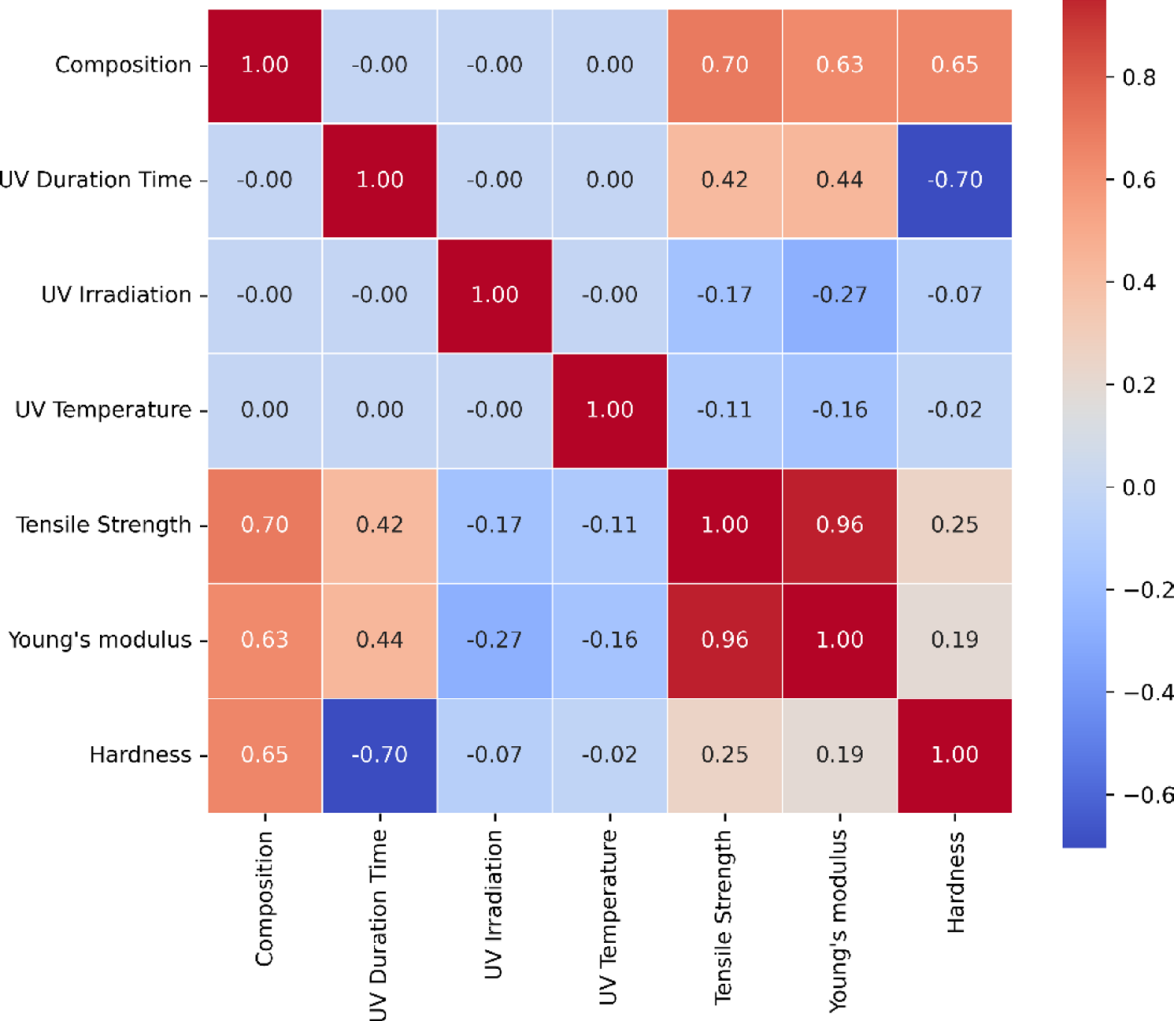
Person Correlation heap map.

The Pearson correlation matrices findings are illustrated in Fig. 23, which show the key processing factors and the mechanical characteristics. It is demonstrated that the composition (wt%) has a significant strong positive association with tensile strength (0.70), Young’s modulus (0.63), and hardness (0.65), demonstrating that increasing composition concentration increases tensile strength, Young’s modulus, and hardness. While UV duration time shows a moderate positive association with tensile strength (0.42) and Young’s modulus (0.44), this indicates that longer UV exposure leads to small improvements in stiffness and strength. However, hardness shows a strong negative correlation with UV duration, as extended UV exposure leads to surface degradation. In contrast, UV irradiation and UV temperature exhibit a significant negative correlation with mechanical characteristics, indicating minimal influence. The tensile strength and Young’s modulus show a high positive correlation (*r* = 0.96), which proves that both strength and stiffness increase. The hardness has a relatively weak positive correlation with tensile strength (0.25) and Young’s modulus (0.19), indicating a distinct surface characteristic. The results show that composition is the dominant parameter for enhancing the material’s mechanical characteristics. In contrast, longer UV exposure primarily degrades surface hardness, while UV Irradiation and UV temperature have minimal effects on mechanical properties.

#### Cross-validation of machine learning models

The findings obtained using the 5-fold cross-validation are presented in Tables 13, 14, and 15. As observed in Table 13, the Gaussian process performed better than ANN and linear regression in predicting tensile strength. The Gaussian process obtained a coefficient of determination R^2^ = 0.99, in addition to MSE = 0.51, RMSE = 0.71, MAE = 0.52, and MAPE = 0.02, achieving close to perfect forecast accuracy. In contrast, ANN and linear regression have higher errors and lower R^2^ scores. The results demonstrate that the Gaussian process is the most accurate model for evaluating tensile strength among the three models. Table 14 presents the Young’s modulus prediction, which follows the same trend and identifies a Gaussian process with better performance, with R^2^ = 0.95, MSE = 55.90, RMSE = 7.47, MAE = 6.41, and MAPE = 0.03. In Table 15 for hardness prediction, R^2^ = 0.98 MSE = 2.09,RMSE = 1.44,MAE = 1.23 and MAPE = 0.01.These findings demonstrate that the Gaussian process leads to the best prediction precision for tensile strength, Young’s modulus, and hardness, compared to ANN and linear regression, as measured by R^2^ and error metrics.

**Table 13:** Model evalauation of tensile strength.

Model	*R*²	MSE	RMSE	MAE	MAPE
Liner Regression	0.70	33.11	5.75	4.49	0.24
ANN	0.99	0.71	0.84	0.65	0.03
Gaussian process	0.99	0.51	0.71	0.52	0.02

**Table 14:** Model evaluation of Young’s modulus.

Model	*R*²	MSE	RMSE	MAE	MAPE
Liner Regression	0.69	378.3	19.45	16.11	0.08
ANN	0.94	63.28	7.95	7.06	0.03
Gaussian process	0.95	55.90	7.47	6.41	0.03

**Table 15:** Model evaluation of Hardness.

Model	*R*²	MSE	RMSE	MAE	MAPE
Liner Regression	0.92	8.27	2.87	2.44	0.03
ANN	0.98	2.23	1.49	1.30	0.01
Gaussian process	0.98	2.09	1.44	1.23	0.01

#### Actual and model comparisons

**Fig. 24:**
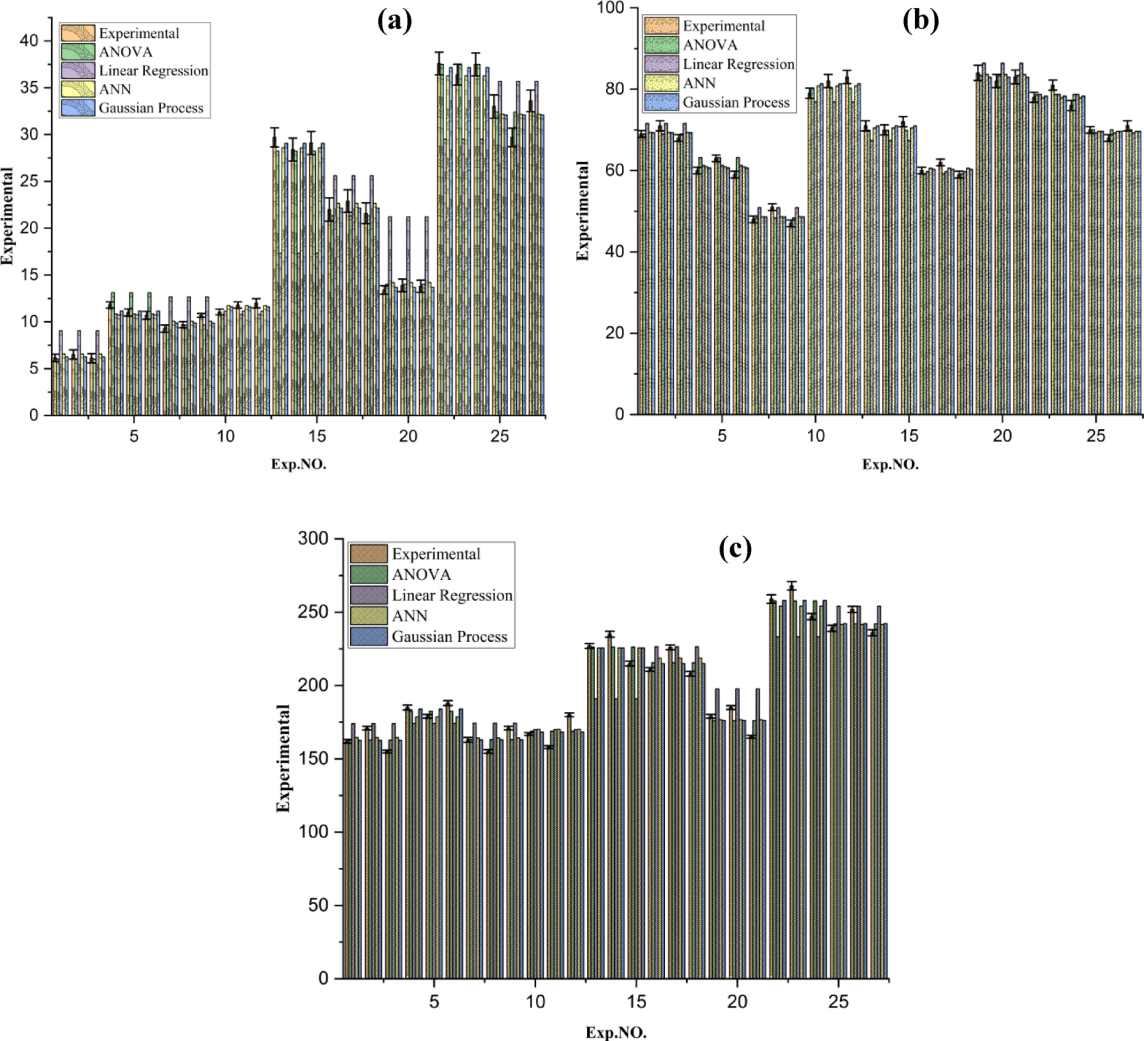
Experimental vs. ANOVA vs. Machine learning models for **a**) tensile strength, **b**)Young’s modulus, **c**) Hardness.

Figure 24(a), (b), and (c) present comparisons of tensile strength, Young’s modulus, and hardness values between experimental results and ANOVA predictions and several machine learning algorithms, such as linear regression, ANN or Gaussian process, for mechanical properties. Figure 24(a), tensile strength ANOVA-predicted values are found to coincide closely with the experimental values, particularly in 1–3 and 7–27 trials, showing that models precisely capture the general trend. Gaussian process and the ANN prediction deliver accurate results through its examination of all experimental results from experiments 1–3, 13–18, and 22–27. The experimental results showed only small differences from the actual outcomes according to the linear regression model. Figure 24(b) contrasts the Young’s modulus between the experimental results and evaluated values through 27 trials. ANOVA outcomes were in close agreement with the experience, particularly for experiments 1–3 and 7–27. machine learning models, Gaussian and ANN models, were superior and more precisely predicted Young’s modulus, especially in experiments 4–9, 12–19, and 20–27. In comparison, linear regression yielded acceptable prediction differences. Figure 24(c) presents the hardness ANOVA results, which closely match the experimental results across all 27 trials. Gaussian and ANN models performed very accurately in predicting hardness, particularly in experiments 1–8,10–21 and 25–27. while linear regression present significant deviation from the experimental results. In summary, Gaussian and ANN have better performance than linear regression. The difference between the experimental results and the predictions of the ANOVA and machine learning models exceeds 10%. demonstrating that these models, especially the Gaussian process and ANN, yield more accurate predictions than linear regression.

## Conclusion

This study systematically investigated the influence of UV exposure on the mechanical properties of polyurethane (PU) reinforced with nanodiamonds (NDs). The experiments were designed using the Taguchi method, and the results were analysed through ANOVA and machine learning models, including linear regression, artificial neural networks (ANN), and Gaussian process regression. The key observations are summarised as follows:

- The incorporation of nanodiamonds significantly enhanced the tensile strength, Young’s modulus, and hardness of the PU matrix.
- It was noted that, 200 h of UV exposure enhances tensile strength and Young’s modulus for all samples, with the most pronounced improvement observed in the 0.5 wt% PU/ND nanocomposite, whereas hardness decreases with increasing exposure due to UV-induced surface degradation.
- ANOVA analyses revealed that nanodiamond composition and UV exposure duration were the primary significant parameters influencing tensile strength, Young’s modulus, and hardness.
- Machine learning models, including ANN and Gaussian process regression, effectively predicted tensile strength, Young’s modulus, and hardness of the samples, with Gaussian regression showing highest accuracy than other models.
- SHAP analysis confirmed that nanodiamond composition and UV exposure duration were the most significant factors governing the mechanical performance of the composites.

These findings demonstrate the strong potential of PU/ND nanocomposites for use in advanced engineering applications, including automotive components, robotic systems, and aerospace structures^44–46^. However, the relatively limited dataset of 27 experimental runs, along with the narrow range of nanodiamond content (up to 0.5 wt%), may restrict the generalization of the developed models. To address this limitation, future work will focus on expanding the dataset and investigating a wider range of filler types and concentrations^47,48^. In addition, the scope of research will be extended to fibre-reinforced composites to enhance model robustness and more fully realise its applicability^49,50^.

## Data Availability

Data is provided within the manuscript.
